# A Review on Surface Stress-Based Miniaturized Piezoresistive SU-8 Polymeric Cantilever Sensors

**DOI:** 10.1007/s40820-018-0189-1

**Published:** 2018-02-02

**Authors:** Ribu Mathew, A. Ravi Sankar

**Affiliations:** School of Electronics Engineering (SENSE), Vellore Institute of Technology (VIT) Chennai, Chennai, Tamil Nadu 600127 India

**Keywords:** SU-8 polymer, Surface stress, Biological sensor, Cantilever, Chemical sensor, Piezoresistor, Immobilization

## Abstract

In the last decade, microelectromechanical systems (MEMS) SU-8 polymeric cantilevers with piezoresistive readout combined with the advances in molecular recognition techniques have found versatile applications, especially in the field of chemical and biological sensing. Compared to conventional solid-state semiconductor-based piezoresistive cantilever sensors, SU-8 polymeric cantilevers have advantages in terms of better sensitivity along with reduced material and fabrication cost. In recent times, numerous researchers have investigated their potential as a sensing platform due to high performance-to-cost ratio of SU-8 polymer-based cantilever sensors. In this article, we critically review the design, fabrication, and performance aspects of surface stress-based piezoresistive SU-8 polymeric cantilever sensors. The evolution of surface stress-based piezoresistive cantilever sensors from solid-state semiconductor materials to polymers, especially SU-8 polymer, is discussed in detail. Theoretical principles of surface stress generation and their application in cantilever sensing technology are also devised. Variants of SU-8 polymeric cantilevers with different composition of materials in cantilever stacks are explained. Furthermore, the interdependence of the material selection, geometrical design parameters, and fabrication process of piezoresistive SU-8 polymeric cantilever sensors and their cumulative impact on the sensor response are also explained in detail. In addition to the design-, fabrication-, and performance-related factors, this article also describes various challenges in engineering SU-8 polymeric cantilevers as a universal sensing platform such as temperature and moisture vulnerability. This review article would serve as a guideline for researchers to understand specifics and functionality of surface stress-based piezoresistive SU-8 cantilever sensors.
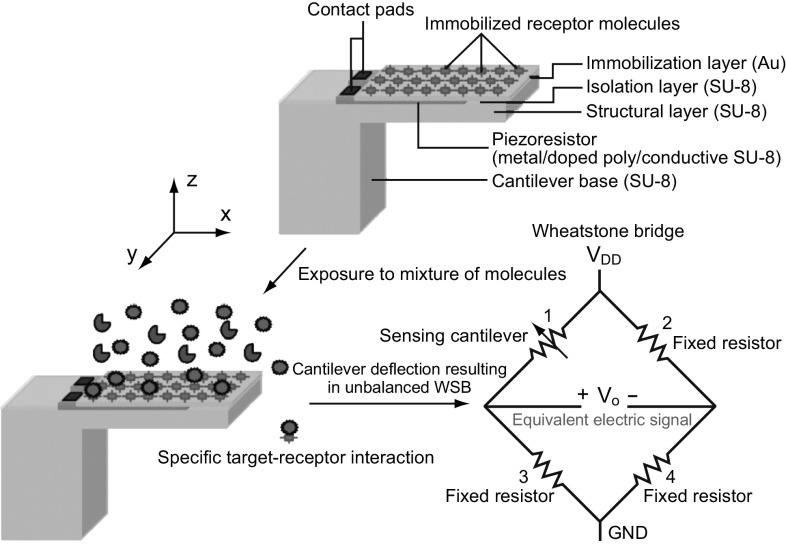

## Highlights

- A critical review on the design, fabrication, and performance aspects of surface stress-based piezoresistive SU-8 polymeric cantilever sensors is presented.
- Specifics of evolution of surface stress-based piezoresistive cantilever sensors from solid-state semiconductor materials to SU-8 polymers are detailed.
- The interdependence of the material selection, geometrical design parameters, and fabrication process of cantilever sensors is explained.
- Challenges which limit the use of SU-8 cantilevers as a universal sensing platform are presented with potential solutions.

## Introduction

Over the years, rapid progress made in micro-/nanoelectromechanical systems (MEMS/NEMS) technology has enabled researchers to develop novel investigation and diagnostic tools, especially in applications related to in situ chemical and biological sensing. This continuous development in the field of MEMS/NEMS has been propelled by the advancements in nano-science and micro-/nano-fabrication technologies [1–11]. MEMS-/NEMS-based cantilever platform sensors have been demonstrated as feasible alternative solutions to the conventional assaying tools due to their advantages of compactness, better sensitivity, lower detection limits, cost-effectiveness, and real-time operation [12]. Typical applications of micro-/nano-cantilever platform-based sensors for chemical and biological sensing include detection of explosives [13], pesticides [14], cancer tissues [15], antibodies [16], heavy metal [17], glucose [18], DNA [19], RNA [20], proteins [21], and viruses [22].

In cantilever-based chemical and biological sensors, target molecules are assayed by converting the mechanical deformation of cantilever platforms into an equivalent electrical signal. In this regard, receptor molecules which have affinity toward target molecules are immobilized on the sensor surface. When exposed to target molecules, target–receptor interactions take place on a sensor surface. These target–receptor interactions induce changes in cantilever characteristics that are in the form of either cantilever displacement (static mode) or change in its resonant frequency (dynamic mode). The aforementioned changes in cantilever characteristics are used to assay the type and quantity of target molecules. Although dynamic mode of measurement depicts higher resolution than static mode, it suffers from fluid damping effects that severely affect sensor sensitivity. In static mode, the measuring cantilever is typically placed in a micro-fluidic chamber with inlet and outlet valves designed in such a way that the fluid flow is laminar, reducing any noise from turbulence. Mechanical stability of the measuring cantilever especially against environmental vibrational noise is ensured by carefully designing the cantilever platform with a high value of resonant frequency. In addition, specific immobilization protocol and proper cleaning techniques reduce cross-sensitivity and biological noise floor. In static mode of operation, conversion of target–receptor interaction-induced deflection of the cantilever platform into an equivalent electrical signal is performed either by optical [23] or by electrical readout methods like piezoelectric [24], piezoresistive [25], and capacitive [26] techniques. Typically, in optics-based readout mechanisms, cantilever deflection is measured with a laser source that is incident on the cantilever surface and a position detector assembly that calibrates the cantilever deflection in terms of shift in laser spot from the initial position. Further, integrated optical readout on waveguides for cantilever sensors is also an alternative option [27]. Such opto-mechanical systems depict high resolution. However, in general optics-based readout methods suffer from limitations due to bulkiness of measurement setup, continuous need for realignment and recalibration, ineffectiveness in opaque medium, complexity in multiplexing, etc. Among the electrical readout techniques, piezoresistive readout is a highly preferred choice due to its advantages like compactness, better scalability, larger dynamic range, possibility of multiplexed operation, independence of operational medium, label-free detection, flexibility of on-chip or off-chip signal processing circuitry, compatibility with integrated circuit (IC) fabrication process flow, to cite a few. The aforementioned factors play a critical role in developing self-sensing, compact, and multi-functional sensors, especially for point-of-care testing (PoCT) and micro-total analysis systems (µTAS).

The micro-cantilever platforms were first utilized as atomic force microscopes (AFMs) for surface imaging applications. The potential application of micro-cantilevers as chemical sensors was first demonstrated by the group led by Prof. Thundat. Since then, in the past two decades, micro-cantilevers have been highly explored as mechanical sensing platforms for assaying various chemical and biological analytes. Initial AFMs were micro-machined on solid-state semiconductors. However, over the years, micro-cantilever-based sensors have been realized with different materials like semiconductor [28–35], metal [36], ceramic [37], plastic/polymer [38, 39], etc. The prime impetus for this endeavor by researchers to find an alternative material for semiconductor was mainly due to the limitation of semiconductor-based devices in terms of their fabrication cost. Among the polymers, SU-8-based piezoresistive cantilever sensors have been demonstrated to have better performance-to-cost ratio than their semiconductor counterparts.

In recent years, reviews on the development and overall performance characterization of cantilevers as sensing platforms have been reported [40–46]. Articles with insights into specific design aspects like enhancement of signal-to-noise ratio (SNR) by improving magnitude of surface stress [47], different immobilization protocols [48–50] are also available. In addition, treatise encompasses examples where researchers have devised the advancements made in polymer micro-machining [51] and polymer MEMS [52]. However, only a handful of researchers [53, 54] have focused on the development- and performance-related aspects of MEMS piezoresistive polymeric cantilevers, especially SU-8 polymer-based sensors. Not only there is a dearth of an article that focuses on the development of SU-8 polymeric piezoresistive cantilever surface stress sensors from their solid-state semiconductor counterparts, but also that provides in-depth specifics of the phenomenon of surface stress generation, details the rationale behind the shift from solid-state semiconductors to polymeric cantilevers, and performs critical examination of variants of SU-8 polymeric piezoresistive cantilevers based on material, design, and fabrication aspects.

In this review article, we critically examine the developments in SU-8 polymer-based piezoresistive cantilever sensors. Primary focus of this review is to provide comprehensive information on the development of piezoresistive SU-8 cantilever sensors with a focus on the design-, fabrication-, and performance-related aspects. Organization of this article is as follows: Sect. 2 details device configuration and working principle of piezoresistive SU-8 polymeric cantilever sensors. Due to their high surface-to-volume ratio, micro-cantilever platforms respond to changes in their own characteristics like mass and spring constant, and thereby to forces even in the range of a few pN. Therefore, cantilever platforms have been extensively explored as mechanical sensing platforms. The cantilever platform-based sensors can be operated in either mass or end point deflection mode. Specifics of basic sensing modes of cantilever sensors are summarized in Sect. 3. Binding of chemical and biological analytes on the cantilever surface induces change in surface stress. Although surface stress-based sensors have found versatile applications, there is no clear understanding on the phenomenon of surface stress generation when target and receptor interact on a surface. The origin, type, and magnitude of surface stress generated due to different target–receptor interactions on cantilever surfaces along with different theoretical and experimental data related to surface stress are detailed in Sect. 4. Due to its origin from AFM, the initial piezoresistive cantilever platform surface stress sensors were based on solid-state semiconductors. The evolution of polymeric cantilevers from their semiconductor counterparts is summarized in Sect. 5. In the last two decades, the piezoresistive element in SU-8 piezoresistive cantilever sensors has been realized with different materials. Specifics of variants of piezoresistive SU-8 polymeric cantilevers which include their classification, structural details, and functional features are explained in Sect. 6. Variants of piezoresistive SU-8 cantilever sensor differ in geometrical design as well as fabrication aspects. The fabrication details of different classes of piezoresistive SU-8 polymeric cantilever sensors covering the fabrication process involved in the same are summarized in Sect. 7. Soon after its inception, SU-8 piezoresistive cantilever sensors have been used as an investigation and detection tool for assaying versatile chemical and biological entities. Typical applications of SU-8 polymeric cantilevers as chemical and biological sensors are summarized in Sect. 8. Finally, in Sect. 9, we discuss the challenges, possible solutions, and future perspectives of SU-8 polymer-based piezoresistive cantilever sensors as the next-generation sensing tool.

## Generic Device Details and Working Principle

To accomplish specific detection of target molecules in a given sample, cantilever-based sensors are operated in either static or dynamic mode. In the dynamic mode, cantilever measures the change in its mass when target–receptor interactions take place on its surface, whereas in the static mode, the addenda of target molecules are assayed by measuring the net cantilever deflection. More specifics of both the sensing modes and the rationale why static mode sensing is preferred over dynamic sensing method will be explained in the later sections of the article.

**Fig. 1:**
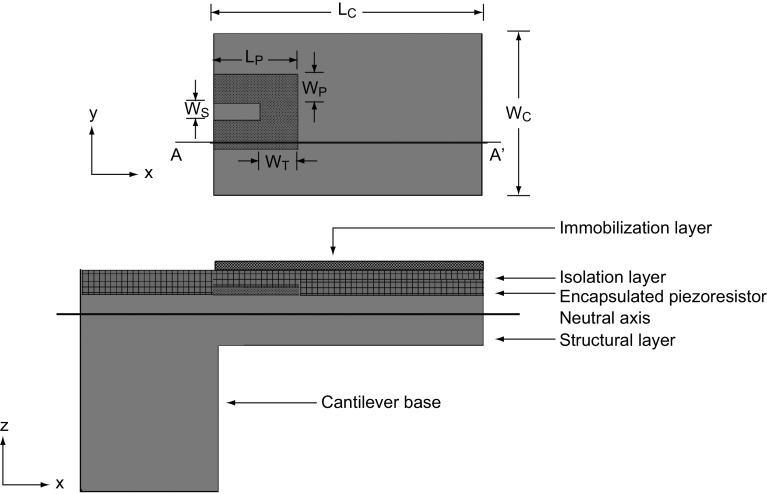
A top view (without the immobilization and isolation layers) and a cross-sectional view (across AA′) of a composite piezoresistive polymeric micro-cantilever sensor. Symbols *L*_C_ and *W*_C_ represent the cantilever length and width, respectively, whereas the symbols *L*_P_, *W*_P_, *W*_T_, and *W*_S_ depict the piezoresistor length, leg width, transverse leg width, and leg space between two piezoresistor strips, respectively

In static mode, SU-8 polymer-based piezoresistive cantilever sensors constitute mainly three components: a mechanical platform, a transduction element, and functional layers. Typical top and cross-sectional views of a piezoresistive SU-8 polymeric cantilever sensor are shown in Fig. 1. The sensor consists of the following layers (from the top): (1) an immobilization layer, (2) an isolation layer, (3) a piezoresistive layer, and (4) a structural layer. For illustration, we have considered a composite slender rectangular cantilever with a U-shaped piezoresistor confined near the central base region of the cantilever. It may be noted that the coverage of piezoresistor on the cantilever and the cantilever platform geometry may vary depending on the piezoresistor material (metal, doped polysilicon, or doped polymer), desired nominal resistance, fabrication processes used to realize the sensor, and a specific application.

When the geometrical features of SU-8 polymeric cantilever sensors (with metal/doped polysilicon/doped composite SU-8 piezoresistors) are compared with solid-state semiconductor cantilevers especially silicon cantilevers with either diffused or ion-implanted piezoresistors reported in the literature the following observations are made: (1) Typically, SU-8 polymeric cantilevers are either three- or four-layered structures (depending on the presence of gold immobilization layer), whereas silicon-based cantilevers are two-layered (without gold) or three-layered (with gold) structures; (2) in SU-8 polymeric cantilevers, the piezoresistor is realized by deposition techniques or spin coating, whereas in silicon cantilevers the piezoresistor is either a diffused or ion-implanted resistor; and (3) in SU-8 polymeric cantilevers, the surface is immobilized by either alkanethiol protocol (gold immobilization layer) or direct chemical modification of SU-8, whereas in silicon cantilevers typically alkanethiol immobilization protocol for gold surface or siloxane immobilization protocol on silicon dioxide surface is performed. On comparing the performance characteristics, both classes depict similar electrical sensitivity with geometrical optimization. Both sensor classes have their characteristic features governed by constituent material set and realization techniques. For instance, silicon cantilever sensors are based on traditional fabrication techniques and can easily be integrated with on-chip signal processing based on CMOS technology. In recent years, continuous improvement in the performance of silicon cantilever sensors has been reported by innovative engineering techniques [55–65]. Silicon cantilevers depict excellent stability against moisture and have better thermal stability. On the other hand, SU-8 polymeric cantilevers show a relatively high performance-to-cost ratio due to low material and fabrication cost. It may be noted that SU-8 polymeric cantilevers show vulnerability toward moisture and temperature variations. However, by controlling the process parameters during sensor realization and by careful sensor module design the vulnerability toward moisture and temperature effects can be reduced as discussed in the later section of this article. Thus, despite various limitations in recent years, there has been much focus on developing SU-8 polymeric cantilever micro-devices for chemical and biological sensing applications.

Structural layer of the cantilever forms the mechanical platform which not only provides mechanical stability to the sensor, but also acts as a stress collector. To perform the electromechanical transduction of structural deformation of the cantilever into an equivalent electrical signal, a piezoresistive layer is deposited atop or doped in the structural layer. Functional layers of the sensor include an isolation layer and an immobilization layer. For reliable operation of the sensor in liquid medium, the piezoresistor is encapsulated by an isolation layer, whereas the cantilever surface is grafted or immobilized with receptors that have high affinity toward the target molecules. Target–receptor interactions on the cantilever surface result in redistribution of energy states on the cantilever surface which is translated into a net cantilever deflection. Even though immobilization of receptors can be performed on the isolation layer, to improve the magnitude of surface stress generated on the cantilever platform, a separate immobilization layer is preferred [50].

**Fig. 2:**
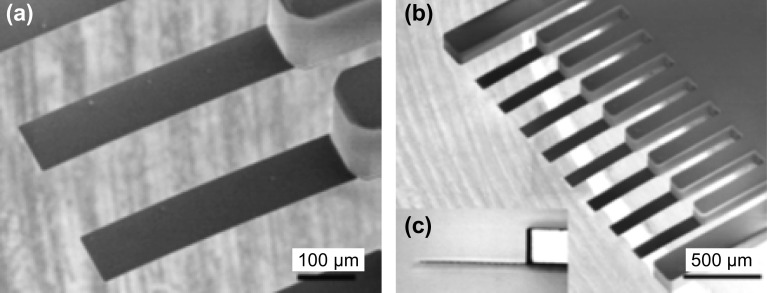
Images of SU-8 cantilever sensor arrays: **a**, **b** dimensions (*L*_C_ × *W*_C_ × *T*_C_) = 300 × 100 × 2 µm^3^, and **c** side view of a SU-8 cantilever. Adopted from Ref. [66]. Copyright (2010) IOP Publishing

**Fig. 3:**
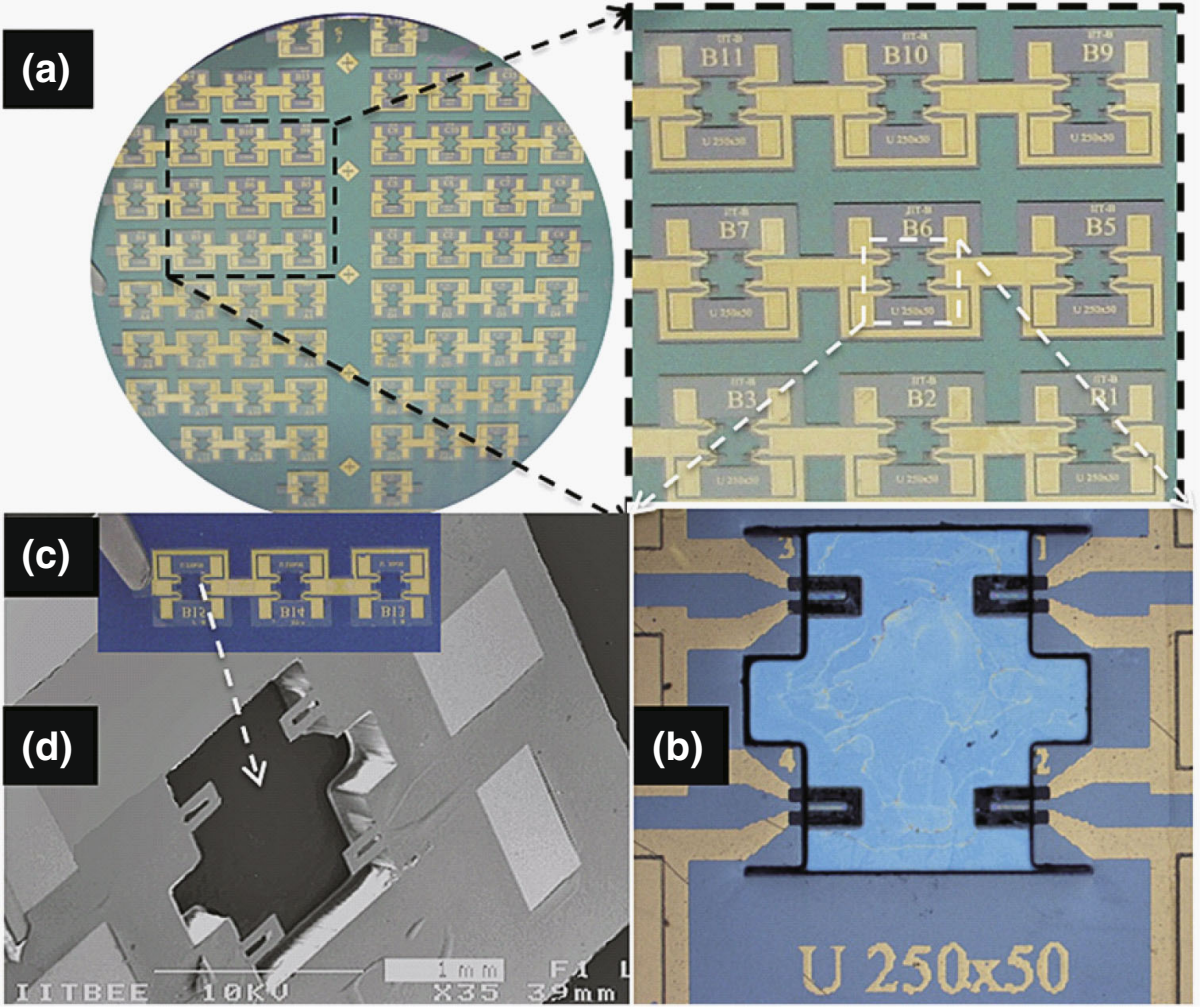
Piezoresistive SU-8 cantilever sensors: **a** an image of processed silicon wafer with a zoom-in view of the sensor device arrays attached to the wafer before release. **b** An image of one of the device chips in the array with four rectangular cantilevers. **c** Array of sensor device chips after the release, and **d** one of the device chips. Adopted from Ref. [67]. Copyright (2011) IOP Publishing

**Fig. 4:**
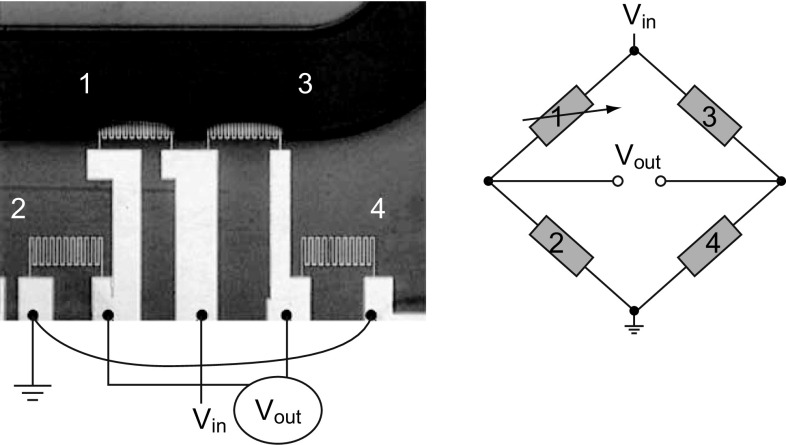
Optical image of a serpentine Au piezoresistor-based SU-8 polymeric cantilever connected in a Wheatstone bridge (WSB) configuration. Adopted from Ref. [68]. Copyright (2005) Elsevier B.V.

In general, SU-8 piezoresistive cantilever sensors are realized using micro-fabrication techniques of spin coating, deposition, photolithography, and etching. At the circuit level, to reduce cross talk and improve signal-to-noise ratio (SNR), piezoresistive SU-8 cantilevers are connected in a Wheatstone bridge (WSB) configuration. A few representative figures of SU-8 cantilevers, image of sensor device chips at wafer level and a close-up view of sensor module of piezoresistive SU-8 cantilevers connected in a WSB configuration are depicted pictorially in Figs. 2, 3, and 4, respectively. Scanning electron microscope images of an SU-8 rectangular cantilever platform, cantilever arrays, and side view of a single cantilever are shown in Fig. 2. Images of an array of batch-fabricated device chips and individual constituent sensor modules of piezoresistive SU-8 cantilevers are shown in Fig. 3. Here, each device chip comprises of four carbon black (CB)-doped SU-8 polymeric cantilevers. An image of SU-8 polymeric cantilever sensor with serpentine-shaped gold piezoresistor is shown in Fig. 4. Typically, serpentine shape of piezoresistors is chosen when metal piezoresistors are used. The rationale behind the premise is to increase the nominal resistance of the piezoresistor. The graphic also represents a WSB-based circuitry, where the measuring cantilever forms one arm of the bridge. Other resistors are formed by the reference cantilever and on-chip resistors.

**Fig. 5:**
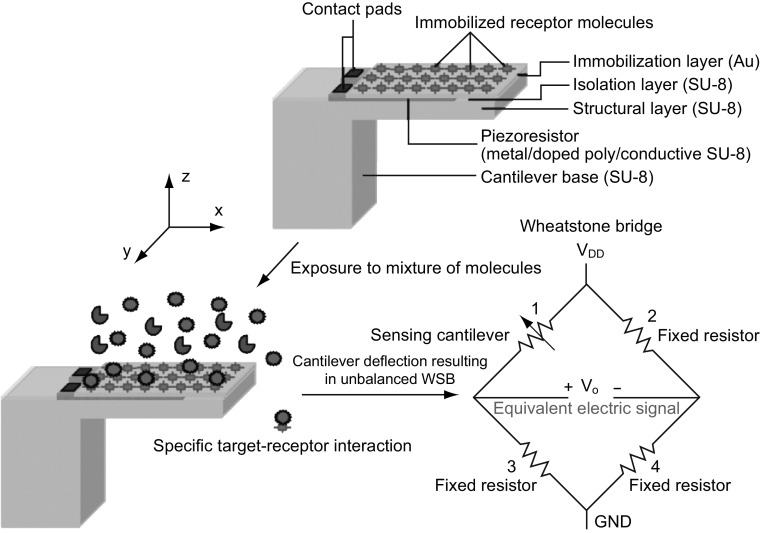
Working principle of piezoresistive SU-8 polymeric micro-/nano-cantilever sensors for chemical/biological sensing applications

**Fig. 6:**
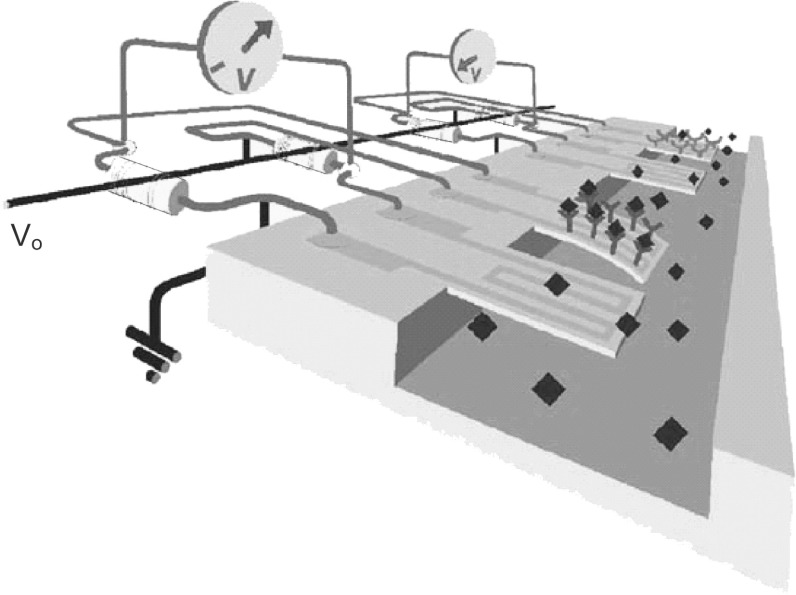
Graphics of a serpentine gold (Au) piezoresistor-based cantilever depicting target–receptor interactions and connected in a Wheatstone bridge (WSB) configuration. Adopted from Ref. [69]. Copyright (2009) Elsevier Ltd.

Detailed functionality of piezoresistive readout-based SU-8 polymeric cantilever chemical/biological sensors is depicted in Fig. 5. The immobilization layer is chemically modified and immobilized with receptor molecules that have high affinity toward target molecules. Immobilization of receptor molecules is performed using techniques like incubation of cantilever arrays in micro-capillaries, micro-contact printing, and inkjet delivery. It is ensured that the immobilization of receptors is only in one surface of the cantilever face on either the top or the bottom. This selective immobilization process ensures generation of differential surface stress. The piezoresistor is typically placed on the region (with respect to neutral plane) where the immobilization surface is present for maximizing electrical sensitivity. When exposed to mixture of molecules, specific target–receptor interactions/bindings result in differential surface stress-induced cantilever bending. The target–receptor bindings result in the generation of either a compressive surface stress or a tensile surface stress leading to either a downward or an upward cantilever bending, respectively. Under mechanical loading, the nominal resistance of the piezoresistor (*R*) placed inside the cantilever stack changes, resulting in either an increase (+ Δ*R*) or a decrease (− Δ*R*) in its value. When the piezoresistive cantilever is placed in one arm of a balanced WSB, with change in its nominal resistance value there is a voltage in the WSB output. The voltage signal is equivalent to the net surface stress generated on the cantilever surface due to target–receptor interactions. A graphical representation of the cantilevers connected in a WSB configuration depicting target–receptor interactions is shown in Fig. 6.

The conversion of target–receptor interactions into an equivalent electrical signal is also possible either by using only the piezoresistive cantilever or by connecting the piezoresistive cantilever along with a fixed resistor in a voltage divider (half-bridge) configuration driven by an excitation source. Although the aforementioned methods are relatively simple to implement, a WSB configuration-based readout method offers advantage in terms of reduced thermal drift sensitivity and nullifying the initial drift in sensor output [70]. Further, it has been found that the temperature drift compensation of a WSB can be improved by a factor of more than two orders of magnitude when a thermally symmetric design is used instead of a conventional WSB configuration. Additional feedback resistors can also be used for temperature compensation. WSB is excited by either a voltage source or a current source with its magnitude limited by power dissipation of the sensor. Typically, output signal from the WSB is conditioned (amplified) using an instrumentation amplifier (INA). Apart from WSB measurement, other measurement techniques have been also reported. For instance, it has been reported that through current excitation of half-bridges, insensitivity against thermoelectric and stray noise can be obtained with a measurement resolution of parts per million (ppm) [71]. Further, the differential amplifier-based measurement instead of WSB has been also reported in the literature [72].

**Fig. 7:**
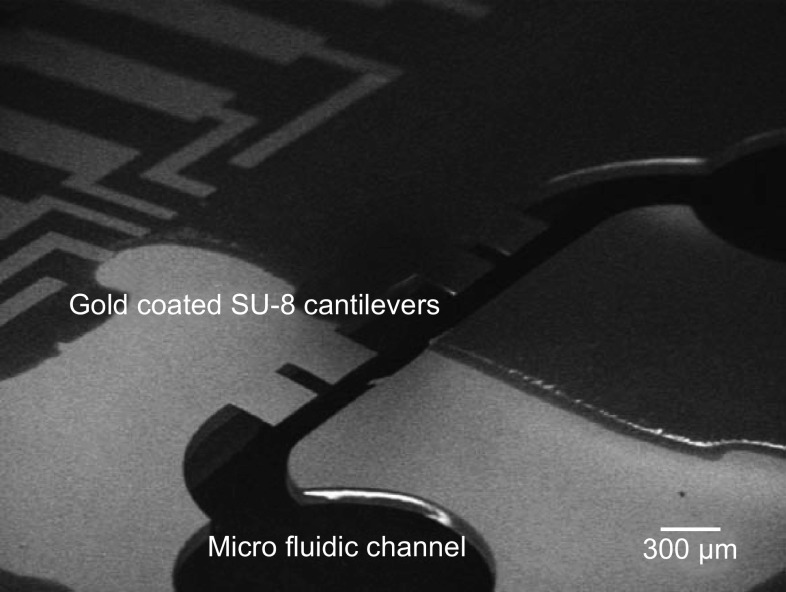
Image of gold-coated SU-8 cantilevers placed in a micro-fluidic channel. Adopted from Ref. [68]. Copyright (2009) Elsevier Ltd.

At the system level, typically for detecting the target molecules especially in the case of biological sensing applications, the cantilevers are placed in a micro-fluidic channel comprising an inlet valve, a channel and an outlet valve as shown in Fig. 7. In chemical sensing systems, the cantilevers are housed in a gas chamber with inlet and outlet valves through which mixture of gas samples are pumped for detection.

**Fig. 8:**
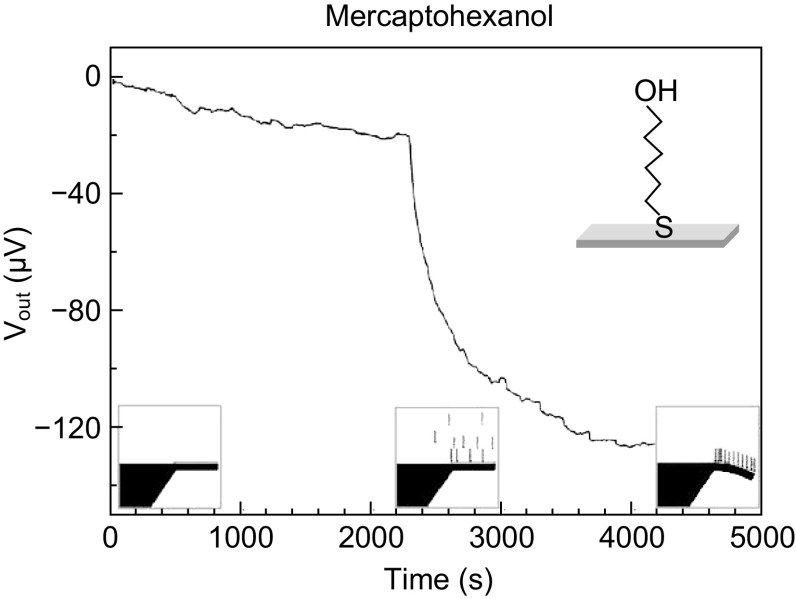
Time response of a piezoresistive cantilever sensor for specific detection of mercaptohexanol. Adopted from Ref. [68]. Copyright (2005) Elsevier B.V.

A typical time response plot of a piezoresistive cantilever sensor depicting various stages of generation of surface stress upon target–receptor interactions as a function of change in voltage is shown in Fig. 8. This particular example depicts the variation in sensor output voltage when specific detection of mercaptohexanol molecules is performed on a gold-coated cantilever immobilized with alkanethiol protocol. There are three stages of sensor response: (1) initial stage: when a stable sensor output is observed due to coating on cantilever surface with self-assembled monolayers (SAMs) of receptors; (2) transition stage: when sensor is exposed to target molecules target–receptor bindings take place on cantilever surface and there is a significant change in sensor terminal voltage; and (3) saturation stage: after the target–receptor pair binding is completed and the change in surface stress saturates, the sensor output becomes constant.

## Sensing Modes

Typically, cantilever sensors are used to assay a specific target molecule or different targets from a mixture of molecules. To accomplish specific target detection, one side of the cantilever is chemically modified with receptors which have high affinity toward the target molecules by an immobilization or grafting process. The specific target–receptor interactions on the cantilever surface can be assayed in terms of either cantilever deflection or change in resonant frequency. In this section, the two operational modes of cantilever sensors are detailed.

### Dynamic Mode

In dynamic mode of operation, change in mass of the cantilever platform due to the addition of target molecules is measured in terms of change in its resonant frequency. The receptor molecules are coated on either one side or both sides of the cantilever platform. The resonant frequency (*f*_0_) of a cantilever platform is mathematically given by Eq. 1 [73]:

1 $$ f_{0} = \frac{1}{2\pi }\sqrt {\frac{{k_{{}} }}{{m_{{}} }}} , $$

where the symbols *k* and *m* represent the flexural rigidity and mass of the cantilever platform, respectively. Resonant frequency of a cantilever is a function of its geometry and constituent material properties. When target molecules bind to the receptors on the cantilever surface, the resonant frequency of the cantilever changes as given by Eq. 2.

2 $$ f_{0}^{*} = \frac{1}{2\pi }\sqrt {\frac{{k_{{}} }}{{m + m^{*} }}} . $$

Change in the resonant frequency depends on the total mass of the target molecules given as m* = *n* × *m*_i_, where *n* is the total number of target molecules and *m*_i_ is the mass of a single target molecule. Shift in the resonant frequency that gives a measure of the target molecules on the cantilever surface is given as $\Delta f = f_{0} - f_{0}^{*}$. In dynamic mode of operation, mass detection resolution as low as yocto-gram (10^−24^ g) has been reported in the literature [74]. Similarly, the typical value of surface stress resolution measured is in the range of 1–4 mN m^−1^ [75]. A measure of the performance of dynamic sensors is its quality factor (Q-factor), which determines the sharpness of resonance peak. Ideally, the Q-factor of a dynamic mode sensor should be infinity for maximizing the minimum detection limits. The Q-factor of a dynamic sensor is reduced mainly due to intrinsic material and extrinsic environmental damping loss [76]. Compared to solid-state semiconductor materials, the material damping loss in polymers is more, which results in reduced Q-factor. Typical values of Q-factor of SU-8 cantilever resonators when operated in air and water are approximately 28 and 1, respectively [77].

**Fig. 9:**
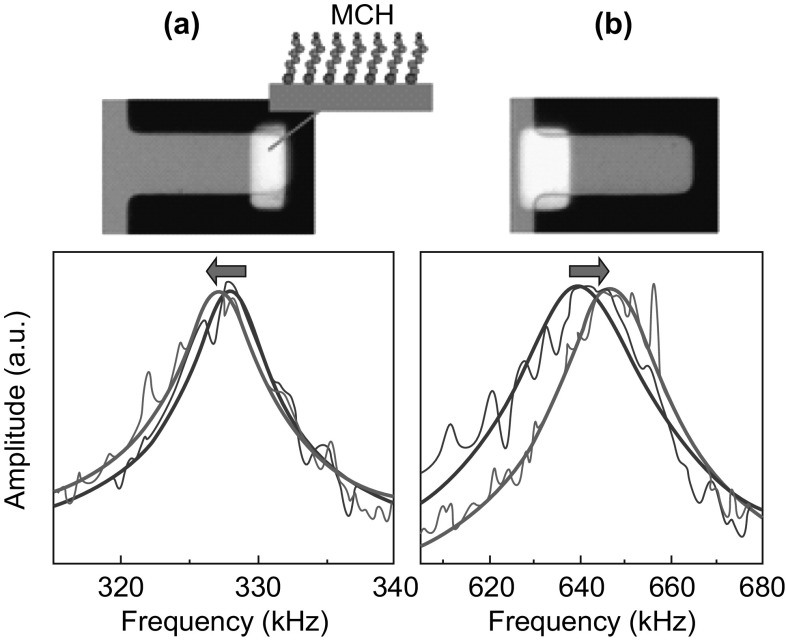
Optical images of cantilevers with selectively coated gold layer coated with self-assembled monolayer (SAM) of mercaptohexanol (MCH). Response of a cantilever operated in dynamic mode: **a** with the gold layer near the free end and **b** with the gold layer near the fixed end. The graphs represent the resonance peaks of the cantilever before (blue line) and after (red line) adsorption of MCH. Adopted from Ref. [81]. Copyright (2009) American Chemical Society. (Color figure online)

Even though high detection resolution is obtained by dynamic mode of sensing, this scheme suffers from limitations such as ineffectiveness in liquid medium due to large fluid damping losses [78], and dependence of resonant frequency shift on the position of target molecule binding site on the cantilever platform [79, 80]. For biological sensing applications, the medium of operation is predominantly liquid. When dynamic sensors are operated in fluids with high viscosity, large fluid/viscous damping results in reduced sensitivity. The positional dependence of shift in the resonant frequency of a cantilever sensor operated in dynamic mode is shown in Fig. 9. When the target–receptor interactions occur near the free end of the cantilever, there is a decrease in resonant frequency, whereas the magnitude of resonant frequency increases when target–receptor interactions takes place near the cantilever fixed end. This is due to the interplay between competing factors of the “mass” and the “flexural rigidity” of the cantilever platform in determining its resonant frequency. When target–receptor interactions occur near the free end of the cantilever, mass effect dominates, resulting in a decrease in the resonant frequency. On the other hand, when the target–receptor bindings occur near the fixed end of the cantilever, flexural rigidity dominates, resulting in a net increase in the magnitude of the resonant frequency. However, due to this dependence of resonant frequency on the position of target–receptor interaction on the cantilever, it is important that in dynamic mode, the cantilever is not fully coated with receptor molecules. This constraint on the coverage area of receptor molecules results in reduced biological sensitivity of the sensor.

### Static Mode

**Fig. 10:**
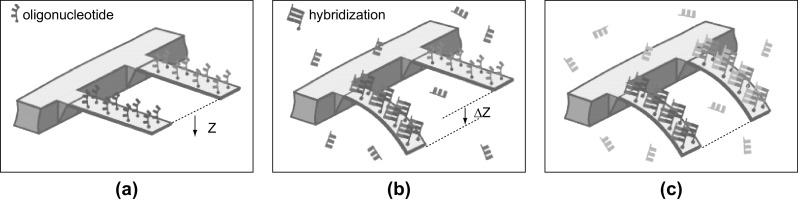
Schematic representation of specific DNA hybridization process and the resultant cantilever deflection. **a** Oligonucleotides with different bases (red and blue) coated on top surface of the cantilevers. Both the cantilevers have net zero displacement. **b** Injection of matching complementary oligonucleotides of base represented in red results in a net deflection of the cantilever due to hybridization. **c** Injection of matching complementary oligonucleotides of base represented in blue results in hybridization-induced deflection. Figures are adopted from Ref. [88]. Copyright (2000) The American Association for the Advancement of Science. (Color figure online)

In static mode of operation, the net cantilever deflection due to target–receptor interactions is measured. Receptor molecules are coated on one side of the cantilever platform. When exposed to target molecules, specific target–receptor bindings on the cantilever cause variation in the surface energy states (surface stress) of the cantilever that are nullified by a net cantilever deflection. Depending on the type of target species, the cantilever bends in either the upward or downward direction [45]. Theoretical computation of the net cantilever displacement corresponding to the difference in the magnitude of surface stress (∆*σ*_s_) between the opposite cantilever faces was first devised by Stoney that is mathematically represented by Eq. 3 [82]:

3 $$ \Delta Z = \frac{{3L_{\text{C}}^{2} (1 - \upsilon )}}{{Et_{\text{C}}^{2} }}\Delta \sigma_{\text{s}} , $$

where ∆*Z* is the cantilever displacement corresponding to the surface stress difference. The symbols *L*_C_, *t*_C_, *υ*, and *E* represent the cantilever length, cantilever thickness, Poisson’s ratio, and Young’s modulus of the cantilever material, respectively. More accurate modeling of surface stress-based cantilever deflections considering the clamping of fixed end of the cantilever was performed by Sader [83]. Comprehensive specifics of surface stress modeling and the response of cantilever platforms under surface stress loading can be found in [84–86]. Typical magnitude of surface stress generated on the cantilever when chemical and biological molecules interact with cantilever surface is in the range of a few mN m^−1^ to a few N m^−1^ that induces cantilever deflection in the range of a few nm to µm, respectively. Ultra-sensitive MEMS cantilever platforms present a viable solution to detect such minuscule forces due to their high surface-to-volume ratio. However, cantilever geometry has to be tailored by careful design (with its flexural rigidity in the range from 0.1 mN m^−1^ to 10 N m^−1^) so that the cantilever is compliant to changes in target–receptor interactions-induced surface stress. The target–receptor interactions result in either an upward or downward deflection of the cantilever. The downward cantilever bending is due to the generation of compressive stress, i.e., decrease in surface energy, whereas the upward cantilever bending is attributed to tensile stress, i.e., increase in surface energy. Using surface stress-based cantilever sensors, deflection sensitivity, minimum detectable deflection, surface stress sensitivity, and minimum detectable surface stress of 0.3 ppm nm^−1^, 4 Å, 3 × 10^−4^ (Nm)^−1^ and 1.4 × 10^−4^ (Nm)^−1^, respectively, have been reported in the literature [87]. An artistic representation of specific target–receptor interactions (DNA hybridization) on cantilevers is shown in Fig. 10 [88]. Figure 10a represents two cantilevers immobilized with two different oligonucleotides or single-strand DNA (ssDNA). The subsequent phase of injection of the complementary DNA strands of oligonucleotides (in red color) is shown in Fig. 10b. Injection and hybridization processes of another set of oligonucleotides (in blue color) are represented in Fig. 10c. Schematic also depicts the downward deflection of the cantilever when DNA hybridization takes place on the cantilever surface. The downward deflection of the cantilever indicates that in this case the surface stress generated due to DNA hybridization is compressive in nature.

A comparison of dynamic and static modes of sensing using a cantilever as the mechanical platform for chemical and biological sensing applications is summarized in Table 1. Compared to static mode of operation, dynamic mode suffers from reduced sensitivity and erroneous measurement due to fluid damping effects. Sensitivity loss due to material damping is prominent, especially in polymeric cantilevers attributed to the high intrinsic material loss of polymers [42]. Even though the performance of cantilevers operated in dynamic mode can be improved by using higher modes of vibration, the reduced amplitude of oscillation makes the readout challenging. In addition, dynamic mode of measurement suffers from the limitation due to stiffness-induced shift in resonant frequency by the adsorbates apart from the mass of target molecules restricting the immobilization area which results in reduced biological sensitivity.

**Table 1:** Comparison of static and dynamic sensing modes of cantilever platforms

Parameters		Sensing modes	
		Dynamic	Static
Sensing principle		Measurement of change in resonant frequency due to change in mass and/or spring constant Measurement of resonant frequency due to change in surface stress	Measurement of cantilever displacement due to change in surface stress
Features		Receptors are immobilized on either one side or both sides of the cantilever Sensitivity can be improved by operating the cantilever at higher modes	Receptors are immobilized on one side of the cantilever Sensitivity can be improved by incorporating stress concentration regions
Limitations		Erroneous due to adsorbate-induced changes in stiffness Susceptible to fluid damping effect Susceptible to material damping effect Dependence of change in resonant frequency on position of the target molecule on the cantilever	Structural nonlinearity due to large deflection of the cantilever Dependence of surface stress generation on immobilization protocols
Suitability for measurement	Liquid	Low	High
	Air	High	High
Resolution		Mass: 10^−24^ g [74]	Cantilever deflection: 4Å [87]

In this regard, static mode of measurement has advantages in terms of reduced dependency of measurement on external ambient and intrinsic material parameters, and better performance in liquid medium which is desirable for chemical and biological sensing applications. When operated in static mode with self-sensing piezoresistive readout, the performance of cantilever can be improved by incorporating stress concentration regions [89–92]. The stress concentration regions (SCRs) act as mechanical amplifiers of stress generated due to cantilever bending, thereby improving electrical sensitivity. In addition, when operated in static mode, polymeric cantilevers depict high displacement sensitivity due to low Young’s modulus of polymers which translates into higher electrical sensitivity. Therefore, compared to dynamic mode, static mode of operation is preferred for polymeric cantilever-based surface stress sensors, especially for chemical and biological sensing applications.

## Theory of Surface Stress

**Fig. 11:**
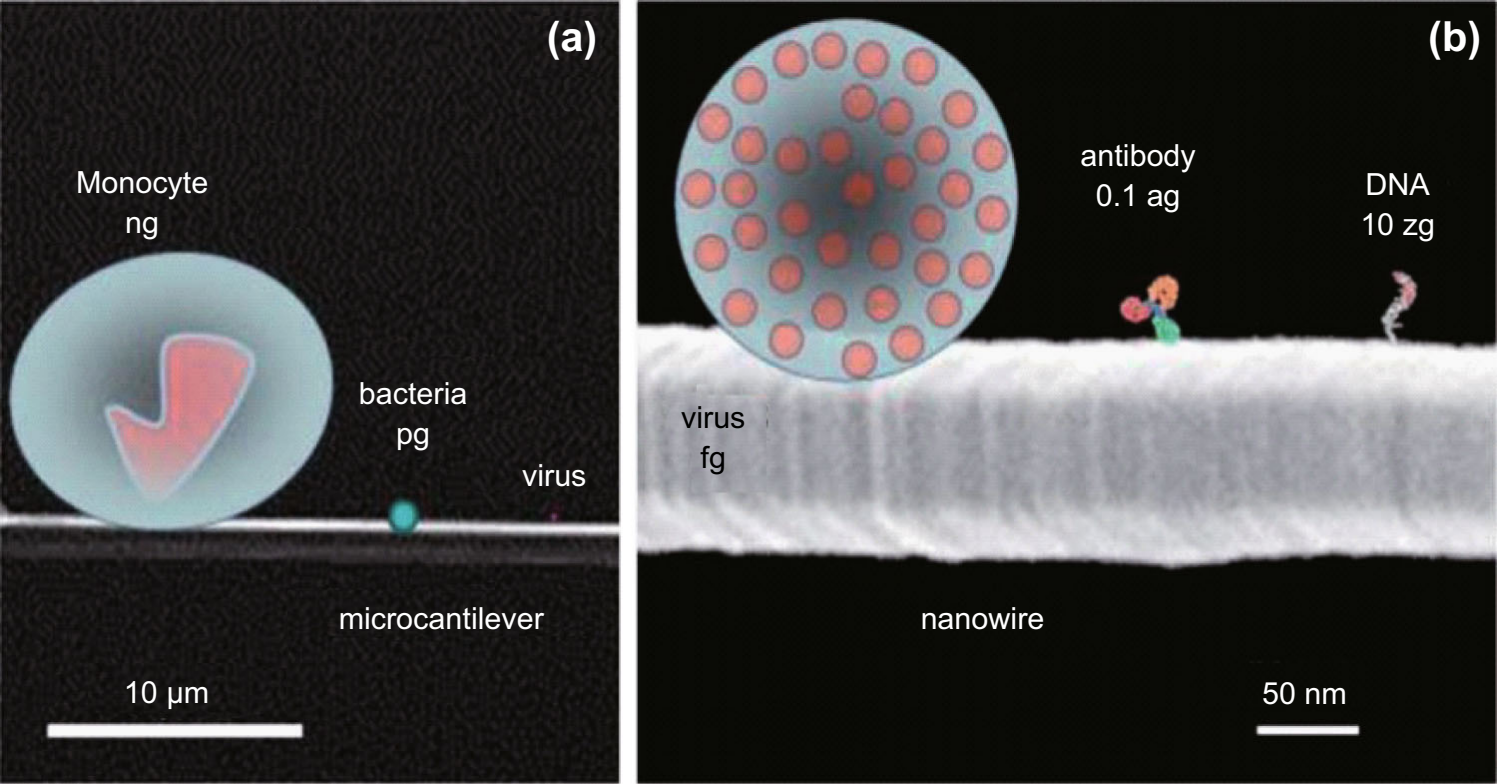
Pictorial representation of different analytes with special emphasis on their size and mass. Adopted from Ref. [44]. Copyright (2012) The Royal Society of Chemistry

The minuscule attractive or repulsive forces that occur on a cantilever surface due to change in its electronic energy states or charge distribution when target–receptor interactions take place on it are known as surface stress. Over the last two decades, more than 50 analytes (chemical and biological molecules) have been assayed using cantilever sensing technology [44]. A graphical representation of the versatility in the size of the analytes assayed is shown in Fig. 11. As evident, the analytes vary not only in terms of their mass, but also in their morphology. Even though surface stress-based cantilever sensors have been widely explored and studied for various applications, the origin of surface stress is still not clear and an in-depth understanding on its basic physics is still to be achieved. In this section, we briefly discuss about various theories proposed by researchers to comprehend the origin of surface stress.

A few researchers have carried out experimental studies to understand the origin of surface stress, and the details are tabulated in Table 2.

**Table 2:** Experimental details of the origin, type, and magnitude of surface stress generated due to different target–receptor interactions on cantilever platform

Reference numbers	Authors	Type and magnitude of surface stress		Cause of surface stress	Target molecule	Readout
[88]	Fritz et al. (2000)	Compressive 5 × 10^−3^ N m^−1^		Electrostatic, steric, and hydrophobic interactions	DNA and protein A immunoglobulin (IgG) (protein–protein) interactions	Optical
[94]	Wu et al. (2001)	Compressive immobilization of ssDNA Tensile with DNA hybridization, but remains net compressive		Configurational entropy and intermolecular energetics (electrostatic and steric interactions)	DNA and biotin–avidin (protein–ligand) binding	Optical
[95]	McKendry et al. (2002)	Compressive 2.7 × 10^−3^ N m^−1^ Single duplex molecule exerts a compressive stress of 1 × 10^12^ N m^−1^		Steric hindrance	DNA	Optical
[96]	Watari et al. (2007)	For pH ≥ 7.0 Compressive 14.5 ± 0.3 × 10^−3^ N m^−1^ For pH < 6.0 Tensile 0.9 ± 0.3 × 10^−3^ N m^−1^		Electrostatic (ionic hydrogen bond interactions, dipole–dipole interactions, and Columbic forces)	Mercaptohexadecanoic acid (MHA) and hexadecanethiol (HDT)	Optical
[97]	Stachowiak et al. (2006)	Compressive 2–32 × 10^−3^ N m^−1^		Steric and hydrostatic hindrances, osmotic and hydration forces	DNA	Optical
[98]	Mertens et al. (2008)	RH: 5–20% Tensile	40–70 × 10^−3^ N m^−1^	Hydration forces Dipole–dipole interactions (Attractive): tensile Steric hindrance (Repulsive): compressive	DNA	Optical
		RH: 50–70% Compressive	150–200 × 10^−3^ N m^−1^			
[99]	Godin et al. (2010)	Compressive 6.3 ± 0.2 N m^−1^		Lennard-John-type interactions (van der Waals and Pauli exclusion): ± 0.001–0.01 N m^−1^ Electrostatic interactions (Coulombic interactions): 0.01–0.1 N m^−1^ (compressive) Changes in electronics charge density of Au surface: 6.3 ± 0.2 N m^−1^ (compressive)	DNA Hexanethiol (C6), octanethiol (C8), decanethiol (C10)	–
[100]	Yang et al. (2011)	TNT, DDT, DNT on Au surface compressive TNT on SiO_2_ tensile TMAH on Au tensile 0–1 N m^−1^		Stereo effect and hydrogen bond intensity	TNT, DDT, DNT, TMAH	Piezoresistive

Even though the concept of surface stress was known from 1900s [93], its application using micro-cantilever structure for sensing was used by Thundat et al. in 1994. Followed by this work on surface stress sensors by the researchers from Thundat et al. group, a few more research articles involving cantilevers were reported by other researchers. Yet, a clear understanding on the origin of surface stress was not reported. Therefore, a few researchers carried out systematic experimental investigation to understand the origin of surface stress. Fritz et al. [88] in the year 2000 were the first to study the origin of surface stress by investigating Watson–Crick base pairing of DNA strands using cantilevers as sensing platform. They have conducted experiments using silicon cantilever coated with gold layer (cantilever dimensions: *L*_C_ × *W*_C_ × *T*_C_ = 500 × 100 × 1 µm^3^, pitch = 250 µm, spring constant = 0.02 N m^−1^, surface coverage: 10 × 10^10^ oligonucleotides per cantilever). They observed a compressive surface stress on Au-immobilized side of a cantilever due to a DNA hybridization process. The origin of surface stress was attributed to electrostatic, steric, and hydrophobic interactions. The electrostatic and steric interactions that were attributed to charge transfer and chain packing density were found to be repulsive in nature, generating a compressive surface stress. The magnitude of compressive surface stress was reported as 5 mN m^−1^ which is equivalent to an actuating force of 300 pN. Subsequently, in the year 2001, Wu et al. [94] reported that the interdependence of configurational entropy changes and intermolecular energetics results in surface stress during DNA hybridization, where the former plays a critical role in determining the direction of cantilever bending. Experiments were conducted with V-shaped silicon nitride cantilevers (V-shaped silicon nitride cantilever coated with Au cantilever details: *L*_C_ × *W*_C_ × *T*_C_ = 200 × 20 × 0.5 µm^3^, Au film thickness = 25 nm with 5-nm chrome for adhesion, surface density of probes: 6 × 10^12^ chains cm^−2^). The group also postulated that the chain length and phosphate buffer (PB) solution concentration also play a vital role in determining cantilever bending. They reported that the immobilization of ssDNA on a cantilever surface generates a net compressive surface stress resulting in downward deflection of the cantilever. However, contrary to the results reported by Fritz et al. [88], their experiments showed that DNA hybridization resulted in tensile surface stress which relieves the compressive stress generated during immobilization process. They also concluded that since the hybridization process always generated tensile surface stress relieving the initial compressive stress, electrostatic and steric interactions are not the only cause of surface stress. The additional factor that resulted in the curvature of cantilevers was proposed as the configurational entropy.

**Fig. 12:**
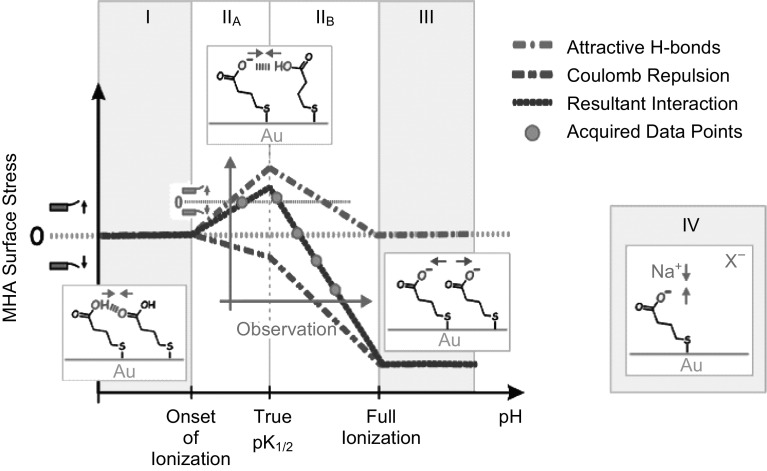
Graphical representation of surface ionization state in different pH regimes. Adopted from Ref. [96]. Copyright (2007) American Chemical Society

Later in 2002, MeKendry et al. [95] demonstrated that a DNA hybridization process generates a compressive surface stress of 2.7 mN m^−1^ on Au-coated cantilevers (rectangular silicon cantilevers coated with 2-nm Ti adhesion layer and 20-nm Au layer, *L*_C_ × *W*_C_ × *T*_C_ = 500 × 100 × 1 µm^3^, pitch 250 µm and spring constant 0.02 N m^−1^). Authors have used high-density probes (1.3 × 10^13^ probes cm^−2^) to measure the type and magnitude of surface stress resulting from hybridization of a single molecule. Their experimental investigation showed that the hybridization of a single molecule results in a compressive stress of 1 × 10^−12^ N m^−1^. They suggested that the electrostatic interactions contribute less to surface stress generation, whereas it is the high-density probes-induced physical steric crowding/steric hindrance effect on Au surface which plays the key role in surface stress generation. In addition, it was demonstrated that surface preparation and DNA probe arrangement on cantilever also have an important role in surface stress generation. Watari et al. [96] performed experiments by immobilizing mercaptohexadecanoic acid (MHA) and hexadecanethiol (HDT) to investigate the nature of surface stress [where the former was immobilized on the sensing cantilevers, whereas self-assembled monolayer (SAM) of the later was grafted on reference cantilevers]. Experiments were conducted with rectangular silicon cantilevers with dimensions *L*_C_ × *W*_C_ × *T*_C_ = 500 × 100 × 0.9 µm^3^ coated with 2-nm Ti adhesion layer and 20-nm Au layer. The group utilized variation in the acid–base properties, i.e., protonation and deprotonation of carboxylic acid-terminated MHA by controlling the pH of the medium. Unlike Fritz et al., who performed their experiments for a fixed pH, Watari et al. demonstrated the importance of pH in governing the nature of surface stress in liquid medium. A graphical representation of the impact of pH variation on surface stress is shown in Fig. 12. Even though the magnitude of surface stress measured by Watari et al. was of the same order of the data published by Fritz for pH > 7.0, for pH 6.0, Fritz et al. reported a compressive surface stress of 2 mN m^−1^, whereas Watari et al. demonstrated a tensile surface stress of − 0.9 ± 0.3 mN m^−1^. This discrepancy was found to be due to the difference in molecular packing and Au morphology during sample preparation. Apart from pH, ionic strength and ionic species present in the aqueous medium were also reported to affect the generation of surface stress. The surface stress generation was proposed to be due to the electrostatic and ionic hydrogen bond interactions between the molecules, and the counter-ions and co-ions present in the medium.

Chemo-mechanics of the transduction of chemical free energy due to DNA hybridization into mechanical deflection of cantilevers was investigated by Stachowiak et al. [97] to understand the origin behind surface stress generation. They conducted experiments with silicon nitride cantilever (with paddle at the end whose dimensions are the following: *L*_C_ × *W*_C_ × *T*_C_ = 200–400 × 30–40 × 0.5 µm^3^ coated with 5-nm Cr adhesion layer and 25-nm Au layer). The group proposed that the surface stress generation is influenced by factors like the length of DNA chain, grafting density, ionic strength of the medium, and hybridization density. Among the aforementioned factors, hybridization density was found to be the dominant factor which combined the effects of chain length and ionic strength in governing the surface stress generation. The surface stress generated due to DNA hybridization was observed to be compressive. It was reported that at a low ionic strength of medium, osmotic pressure of counter-ions prevails over intermolecular forces, whereas at high ionic strength, hydration forces dominate. Mertens et al. [98] proposed that the cause of surface stress in highly packed SAM-based DNA hybridization process is the steric and hydration forces along with steric crowding effects. Experiments were conducted with silicon cantilever coated with gold (silicon micro-cantilever with gold coating of typical dimensions: *L*_C_ × *W*_C_ × *T*_C_ = 400 × 100 × 0.6 µm^3^, coated with 2-nm Cr adhesion layer and 20-nm Au layer, resonant frequency: 5.3 ± 0.1 kHz, and spring constant: 0.029 ± 0.001 N m^−1^). Hydration/dehydration cyclic tests were performed to investigate the effect of RH on surface stress generation for immobilized ssDNA on a cantilever surface. It was demonstrated that the variation in RH affects not only the magnitude of surface stress, but also its type. Results depicted that for RH in the range from 5 to 20%, the surface stress was tensile with its magnitude ranging from 40 to 70 mN m^−1^, whereas an increase in RH (50–70%) resulted in a large compressive surface stress (150–200 mN m^−1^). It was proposed that the tensile and compressive surface stress generated on the cantilever is due to the attractive dipole–dipole interactions and repulsive steric hindrance, respectively. While the change in surface stress induced by ssDNA was found to be affected by various parameters, in hybridized DNA the specific Watson–Crick base binding (intermolecular interaction) mainly resulted in surface stress changes. The competing factors of hydrogen bonding (tensile stress) and steric hindrance interactions (compressive stress) were found to play a less significant role in surface stress generation.

**Fig. 13:**
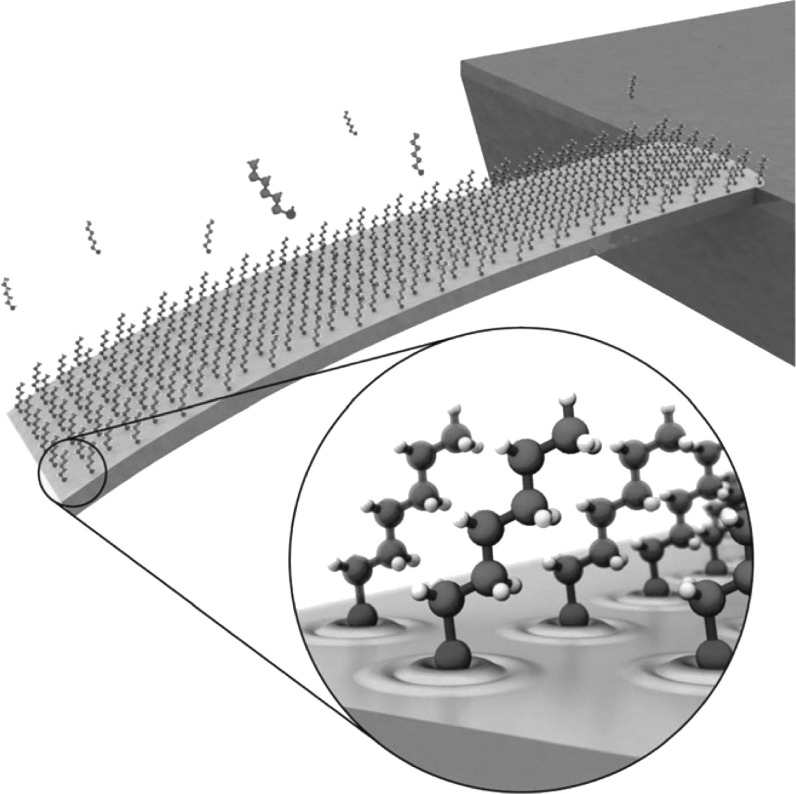
Pictorial representation of alkanethiol-based self-assembled monolayers on a gold-coated cantilever platform and a zoom-in view of the redistribution of electronic states of Au immobilization layer. Adopted from Ref. [99]. Copyright (2010) IOP Publishing

A detailed investigation on the contribution of various factors that affect surface stress generation in Au-coated cantilevers was performed by Godin et al. [99]. They proposed that surface stress is due to three reasons: (1) Lennard-John’s interactions between adsorbed molecules which can be due to either van der Waals forces (attractive) or Pauli exclusion forces (repulsive), (2) electrostatic interactions between Au–thiol bonds, and (3) changes in electronic state of the surface that results in net charge redistribution on the cantilever surface during a DNA hybridization process. However, the group reported that among the three factors, the large compressive stress generated in DNA hybridization process is largely due to the change in electronic state of the underlying Au immobilization surface. A pictorial representation of the immobilized gold-coated cantilever with self-assembled monolayers of receptors and modified electronic energy states is shown in Fig. 13. The redistribution of energy states of Au surface due to Au^+^S^−^ bond and charge transfer from Au surface to S atom reduces the bond strength of Au surface atoms, resulting in the generation of compressive surface stress. In addition, unlike previous studies, they reported that the generation of surface stress is independent of molecular chain length. This discrepancy between the reported results in previous studies was attributed to the dependence of surface stress generation on the grain size of Au immobilization surface. More recently, Yang et al. [100] reported that the origin of surface stress is due to interface vertical effects and lateral interactions. They had carried out experiments with rectangular silicon dioxide cantilever with a thin U-shaped SCS piezoresistor, silicon dioxide insulating layer, and immobilization layer realized with thin film of Au (cantilever dimensions: *L*_C_ × *W*_C_ × *T*_C_ = 90 × 20 × 1.0 µm^3^). Interface vertical effects include interfacial energy change and charge redistribution, whereas van der Waals force, electrostatic Coulombic effect, intermolecular hydrogen bond intensity, and steric effects contribute to lateral interactions. However, lateral interactions were found to play a more significant role in generating surface stress. Among the factors which contribute to the lateral interactions, the intermolecular hydrogen bond intensity and steric interactions were reported to be dominating over the other two factors.

Thus, the reported results show that the origin of surface stress is not due to a single factor, but due to the complex interchange of energies attributed to various parameters like electrostatic interactions, steric interactions, hydrophobic interactions, configurational entropy, hydration forces, Lennard-John’s interactions, changes in electronic charge density of Au layer, stereo effect, and hydrogen bond density. The temporal variation of surface stress from the initial immobilization of receptors to various stages of target–receptor interactions is a function of factors like pH, RH, temperature, receptor coverage, chain length (in case of DNA), ionic concentration of medium, characteristics of the immobilization surface, position of immobilization surface, and size of target molecules.

Active research is underway to explore the possibility of direct immobilization of receptors without the gold immobilization layer. For instance, in the case of SU-8 cantilevers, the top isolation layer realized with a thin layer of SU-8 can also act as the immobilization surface. Apart from cost reduction, direct immobilization on SU-8 polymer is seen as a possibility to reduce high-temperature process-induced thermal stress during separate Au metal deposition on SU-8. Covalent bond-based immobilization techniques on polymer surface have been reported by immobilization of functional groups like CHO, SH, NH_2_, etc. One of the techniques used to immobilize amino functional groups on the SU-8 polymer is aminosalinization process, which takes advantage of the opened epoxied groups of SU-8 polymer [101]. Other techniques include treatment of SU-8 with glycine [102], silane and polyethylene glycol (PEG) [103], and ceric ammonium nitride (CAN) [104, 105]. Using surface modification techniques, ssDNA probe density of 100 fmol mm^−2^ has been reported in the literature [106]. For more details regarding the various immobilization protocols, different immobilization surfaces, surface stress enhancement techniques, etc., readers may refer the literature [48–50].

As discussed earlier, covalent immobilization protocol is the most stable protocol. SU-8 cantilevers support covalent bond only when functional groups like amine, aldehyde, thiol, and carboxyl are present. Immobilization of receptors is accomplished by either a wet or a dry method. Literature encompasses examples of both the wet and dry immobilization techniques. A brief summary of wet and dry methods used for surface treatment is detailed in Table 3. Typically, wet method of surface modification has been reported, in which acid/base chemicals are used for surface modification. However, wet method of surface modification suffers from the following limitations: (1) It uses strong oxidizing/hydrolyzing agents that damage device surface apart from the immobilization region, (2) it requires repetitive steps which involves immersion, washing, and drying the surface which is time-consuming and results in surface damage or even increases chance of contamination, and (3) it requires tight control over process parameters and ambient conditions like temperature and pH value of the medium. Dry surface modification is performed in several ways. For instant, by exposing the surface to UV light in ammonia (environment), amine group is immobilized. Another method is by using oxygen/ammonia plasma treatment by which hydroxyl/amine groups are immobilized on SU-8 surface. However, surface modification using exposure to plasma damages the device surface, and grafting using UV light is not only time–consuming, but also results in change in the material properties of polymer. One alternative dry immobilization technique is by using the pyrolytic dissociation of ammonia for grafting amine groups in a hotwire chemical vapor deposition (HWCVD) environment. Compared to the wet method, the dry method holds the following advantages: (1) Damage to the sensor surface and alterations to constituent material properties are negligible especially in the technique based on HWCVD due to low temperature requirement, and (2) unlike typical wet grafting methods, the use of strong chemical is avoided, thereby resulting in unaltered surface properties. Retaining the surface characteristics of devices becomes important especially when the device is reused, i.e., for device repeatability or reusability.

**Table 3:** Details of various dry and wet immobilization techniques with their respective application

Authors and year	Immobilization method	Immobilized group/process	Application and device
Mayer et al. (2003) [107]	Dry (plasma)	Functionalization of amino group	Protein detection
Gao et al. (2006) [108]	Wet	Photopolymerization Surface graft polymerization	Hydrogel detection with potential SU-8 micro-channels
Wang et al. (2007) [105]	Wet	Surface graft polymerization	Mouse IgG detection with SU-8 micro-cantilever and micro-channels
Joishi et al. (2007) [109]	Dry	Aminosilanization	Human immunoglobulin (HIgG) detection using SU-8 micro-cantilevers
Blagoi et al. (2008) [110]	Wet	Aminosilanization	Goat anti-mouse antibody whole-molecule detection with SU-8 micro-wells
Deepu et al. (2009) [102]	Wet	Carbodiimide/succinimide	Human immunoglobulin G (HIgG) detection
Cao et al. (2011) [111]	Wet	Covalent bonding of Au nanoparticles	DNA hybridization with micro-device

The covalent bonds formed especially in the case of thiol–gold are strong with a binding energy of 120 kJ mol^−1^ [112]. Thus, it becomes difficult to dissociate the covalent bond without additional energy. Premise presents a challenge in terms of repeatability of devices. However, when external energy is provided covalent bonds dissociate and this may be used for refreshment of cantilever surface. Typically, these refreshment techniques are based on light and temperature. In optics-based refreshment technique, light energy incident from a light source dissociates the bond, whereas in temperature-based refreshment techniques the rise in surface temperature of device typically by an integrated heater resistor results in dissociation of covalent bonds. In piezoresistive readout-based cantilever sensors, refreshment using an integrated heater becomes a more attractive option since the piezoresistor and the heater can be realized using similar steps. In addition, an integrated method provides compactness to the device, thereby reducing device footprint. The integrated heater technique uses joule heating to increase the sensor surface temperature for refreshment. Integrating a heater element can influence the detection technique and piezoresistance properties. For instance, the sensor reported for detection of TNT vapors in [113] uses the heat generated by the in-built heater resistor for deflagration of TNT vapors, resulting in enhanced heat generation and thereby cantilever bending. The resultant cantilever bending due to the heat generated by deflagration of TNT vapors is gauged by the piezoresistor. Although integrated heater-based sensor design has several advantages, it may result in variation in piezoresistor properties due to temperature coefficient of piezoresistance, irreversible plastic deformation, and thermal drift in device output. Cleaning the device surface once the bond dissociates is performed by chemical or dry methods. The extent of cleaning and surface regeneration by subsequent chemical modification determines the immobilization efficiency and therefore biological sensitivity of devices.

## Evolution: Solid-State Semiconductor to Polymeric Cantilevers

Due to its origin from the matured microelectronics technology, initial MEMS cantilever sensors were based on solid-state semiconductor materials, especially silicon and its derivatives. A timeline of the evolution of cantilever platform sensors is shown in Table 4. The timeline includes representative papers of different cantilever sensors, and omission of any important references is regretted. Miniaturized cantilevers with their dimensions in micro-regime were first used as atomic force microscopes (AFMs) for surface imaging applications by Binning et al. [114]. The reported AFM consisted of a slender rectangular cantilever with a sharp tip at its end that allowed topological measurement of a sample surface with atomistic resolution. In 1991, Thundat et al. [115] used AFM for imaging deoxyribonucleic acid (DNA) strands at different levels of relative humidity. In the next few years, the same group explored the use of AFM in understanding both single and double strands of DNA in various external conditions [116–119]. Gimzewski et al. [120] in 1993 demonstrated a micro-cantilever-based chemical sensor to detect the catalytic conversion of hydrogen (H_2_) and oxygen (O_2_) into water (H_2_O). The sensor constituted a silicon cantilever coated with a thick layer of aluminum (Al) metal and a thin film of platinum (Pt) atop which the reaction takes place. This multi-morph configuration could convert the heat flux generated from the catalytic reaction into cantilever deflection due to the difference in temperature coefficient of expansion (TCE) of two layers up to 1 nW with a response time of 1 ms. Subsequently, in 1994 several concurrent developments demonstrated the bimetallic configuration of a cantilever platform coated with a metal layer as a viable sensing platform. For instance, Barnes et al. developed a bilayer of silicon nitride cantilever and a thin film of aluminum atop as a calorimeter that depicted sensitivity as low as 10 pW [121]. By the mid-1990s, MEMS-based cantilever platforms were demonstrated as physical and chemical sensors by Thundat et al. The group used micro-cantilevers with metal layers for the detection of surrounding humidity (bilayer of silicon/silicon nitride cantilevers coated with a layer of gold and/or aluminum) [122] and mercury vapors [123]. Further in the year 1995, Raiteri et al. [124] demonstrated the applicability of AFM (silicon nitride cantilevers) coated with gold/platinum metal layer in measuring electrochemically induced surface stress using optical leverage technique. These cantilever platforms exhibited ultra-high sensitivity to changes in their external environment and their own mass mainly due to their large surface-to-volume ratio. In addition, the micro-cantilevers had the inherent advantage of low spring constant and high resonant frequency, thus making them highly sensitive to external forces. Later, this arrangement of micro-cantilever platform with a metal layer was demonstrated as a viable biological sensing tool for applications like detection of protein [125] and DNA [88] by various researchers.

**Table 4:** Chronological details of the evolution of micro-/nano-cantilever sensors

Authors and year	Material set	Constituent layers	Readout technique	Comments
Binning et al. (1986)	Cantilever Au, tip diamond	Structural layer: Au	Tunneling current	AFM topological measurement
Gimzewski et al. (1993)	Cantilever Si, Al + Pt coating	Additional layer: Al + Pt	Optical	Calorimeter-based chemical sensing
Thundat et al. (1994)	Cantilever Si/Si_3_N_4_ + Au/Al coating	Additional layer: Au/Al Structural layer: Si/Si_3_N_4_	Optical	Humidity and mercury vapor sensing
Raiteri et al. (1995)	Cantilever Si_3_N_4_ + Au/Pt coating	Structural layer: Si_3_N_4_ Additional layer: Au/Pt	Optical	Measurement of electrochemically induced surface stress
Boisen et al. (2000)	Cantilever Si, piezoresistor doped Si	Immobilization layer: gold/polymer Protective layer: SiO_2_ Piezoresistive layer: p-poly-Si Isolation layer: SiO_2_ Structural layer: Si	Piezoresistive	Temperature, humidity, and alcohol sensing
Thaysen et al. (2002)	Cantilever SU-8, piezoresistor Au	Immobilization + isolation layer: SU-8, Piezoresistor layer: Au Structural layer: SU-8	Piezoresistive	Surface micro-machining
Rasmussen et al. (2003)	Cantilever Si_3_N_4_, piezoresistor doped polysilicon	Immobilization layer: Au Isolation layer: SiN_*x*_ Piezoresistor layer: p-poly-Si, Structural layer: SiN_*x*_	Piezoresistive	ssDNA sensing Bulk + surface micro-machining
Gammelgaard et al. (2006)	Cantilever SU-8, piezoresistor CB SU-8	Isolation layer: SU-8 Piezoresistor layer: CB SU-8 Structural layer: SU-8	Piezoresistive	Surface micro-machining
Zuo et al. (2006)	Cantilever SiO_2_, piezoresistor p-SCS	Immobilization layer: Au Isolation layer: SiO_2_ Piezoresistor layer: p-SCS Structural layer: SiO_2_	Piezoresistive	Methyl-phosphonate sensing Bulk micro-machining
Kale et al. (2009)	Cantilever SU-8, piezoresistor p-poly-Si	Immobilization + isolation layer: SU-8 Piezoresistive layer: p-poly-Si, Structural layer: SU-8	Piezoresistive	Surface micro-machining, HWCVD
Seena et al. (2009)	Cantilever SU-8, piezoresistor CB SU-8	Immobilization + isolation layer: SU-8 Piezoresistive layer: CB SU-8 Structural layer: SU-8	Piezoresistive	Surface micro-machining
Reddy et al. (2012)	Cantilever SU-8, piezoresistor CB SU-8	Immobilization + isolation layer: SU-8 Piezoresistive layer: CB SU-8 Structural layer: SU-8	Piezoresistive	CO sensing Surface micro-machining
Patil et al. (2014)	Cantilever SU-8, piezoresis or CB SU-8	Immobilization + isolation layer: SU-8 Piezoresistor layer: CB SU-8 Structural layer: SU-8 Prohibition layer: Au	Piezoresistive	Soil moisture and relative humidity (RH) sensing Surface micro-machining

The initial AFMs and cantilever sensors were either optics- or resonant frequency shift readout-based systems (where, in the former technique, a laser beam is incident at the apex of the cantilever and its shift in position is measured with a photodetector, and in the latter, the shift in the resonant frequency of the cantilever is measured using a piezoelectric actuation system). Even though optics- and resonant frequency shift-based readout techniques exhibited cantilever displacement resolution in nanometer (nm) [126] and mass detection sensitivity in picogram (pg) [127] range, their applicability was restricted to vacuum and air operational medium mainly due to the following reasons: (1) inaccurate measurement in liquid medium due to fluid damping effect, (2) ineffectiveness in opaque liquid, (3) bulkiness of measurement setup, and (4) need for continuous realignment and recalibration. To overcome the limitations of optics- and resonant frequency-based readout methods, self-sensing piezoresistive readout technique was adopted by various researchers in the mid-1990s. Piezoresistive readout was first demonstrated by Tortonese et al. in 1991 [128] in AFM cantilevers. Even though other integrated readout techniques like capacitive [129], piezoelectric [130], tunneling [131], and integrated optical waveguide [132] were also implemented, piezoresistive readout demonstrated better performance. A few early illustrations of piezoresistive cantilever-based biological and chemical sensors include analysis of the dehydration of copper sulfate pentahydrate with picogram resolution [133], detection of alcohol vapor with detection limits below 10 ppm [134], measurement of temperature, humidity and alcohol with a minimum detectable cantilever deflection of 1 Å and a deflection sensitivity of 1.6 nm (µW)^−1^ [135], investigation of surface stress due to self-assembled alkanethiol on gold surface [136]. Furthermore, to reduce the probability of non-specific detection, specific receptors, which have high affinity toward target molecules, were immobilized on the cantilever surface. The specific bindings of target molecules on the cantilever platform resulted in either a change in its mass or a variation in its surface energies that resulted in cantilever deflection.

In the past decade, various solid-state semiconductor cantilever sensors based on silicon [137–142], silicon nitride [143–146], and silicon dioxide [147–153] with integrated doped single crystalline, polysilicon, and metal piezoresistors have been demonstrated. Typically, in solid-state semiconductor-based piezoresistive cantilever sensors, the structural layer is realized with materials like silicon, silicon dioxide, or silicon nitride and the piezoresistor element is doped silicon, doped polysilicon, or gold. Even though semiconductor cantilever sensors have advantages in terms of low cost due to batch fabrication (when produced in large volume) and performance, the research and development to realize such sensors is cumbersome and limited due to the stringent requirement of clean room facilities and large initial investments for equipments. This was the impetus for researchers to find an alternative material, which could match semiconductor-based sensors in terms of performance with a reduction in material and fabrication cost.

Although various materials like metal, silicon carbide, graphene, diamond, ceramic, etc., have been used to realize MEMS-based devices, their applicability to realize piezoresistive cantilever sensors is limited due to higher stiffness of the structure, high material cost, fabrication complexity, and incompatibility with batch fabrication. Polymers were considered as alternative materials due to their low Young’s modulus, biocompatibility, and cost-effectiveness in terms of both material and fabrication. Pechmann et al., in 1994, were the first to report polymeric cantilever devices based on novolak photoresist [154]. Since then, various polymers such as parylene [155], polypropylene [156], fluoropolymer [157], SU-8 [158], polyethylene terephthalate [159], polyimide [160], TOPAS^®^ [161], polystyrene [162], polydimethylsiloxane (PDMS) [163], and polymethyl methacrylate (PMMA) [164] have been extensively explored to realize miniaturized devices. An overview of material properties, fabrication process, and representative applications of the aforementioned polymers in MEMS are summarized in Table 5.

**Table 5:** Overview of various polymers with their representative fabrication process, features, and applications in MEMS

Polymer	Fabrication process	Features	Applications
Parylene	Chemical vapor deposition (CVD) Etching by oxygen plasma Hot embossing Lithography	Young’s modulus, *E* ~ 5 GPa Chemically inert Low intrinsic stress and gas permeability Hydrophobic Optically transparent Vulnerable to temperature	Electrostatic actuator [155] Micro-valve [165] Spring [166] Electrostatic micro-peristaltic pump [167]
Polypropylene	Injection molding Laser ablation	Young’s modulus, *E* ~ 1.45 GPa Vulnerable to oxidants Thermal resistance Large thermal coefficient of expansion Opaque	Surface stress cantilever sensor [156] Component in air-coupled piezoelectric transducer [168] Piezo-electret film transducer [169]
Fluoropolymer Teflon^®^ Polytetrafluoroethylene Tefzel^®^ Fluoroethylenepropylene	Spin coating Ion beam sputter etching Magnetically controlled reactive ion etching	Young’s modulus, *E* ~ 1.45 GPa (Teflon^®^) Chemically inert Hydrophobic Thermally stable Teflon^®^ smoothest surface morphology	AFM-based biochemical sensor [157] Micro-tube [170] Micro-fluidic channel [171]
SU-8	Spin coating Photolithography Excimer laser patterning Pyrolysis Dry etching	Young’s modulus, *E* ~ 5 GPa Low molecular weight Chemically inert High refractive index Compatibility with grayscale lithography	Optical waveguide [172] Micro-needles [173] Micro-resonator [174] AFM cantilever [175] Surface stress cantilever sensor [68]
Polyethylene terephthalate	Excimer laser patterning and laser ablation	Young’s modulus, *E* ~ 2.8 GPa Excellent resistance to moisture High impact resistance	Cantilever biosensor [159] Mechanical substrate [176] Micro-pump [177]
Polyimide	Spin coating Dry etching using oxygen or fluorine plasma Hot embossing Lithography	Young’s modulus, *E* ~ 7.5 GPa Chemically inert Stable at high temperature and heat Vulnerable to alkalis Low susceptibility to moisture	Scanning probe [160] Tactile sensor [178, 179] Humidity sensor [180] Micro-channels [181]
TOPAS^®^	Spin coating Nano-imprint lithography	Young’s modulus, *E* ~ 3.5 GPa High chemical inertness Low susceptibility to moisture Good optical transmission	Water vapor sensor [161] Optical waveguide [182] Micro-fluidic devices [183]
Polystyrene	Injection molding Solvent casting technique	Young’s modulus, *E* ~ 3.0 GPa Vulnerable to moisture Optically transparent	Surface stress sensor [162] Accelerometer [184]
PDMS	Spin coating Cast molding	Young’s modulus, *E* ~ 0.75 MPa Incompatible with organic solvents Optically transparent Gas permeable	Micro-valve [185] Magnetic actuator [186] Micro-pump [187] Micro-channel [188]
PMMA	Injection molding Hot embossing Wire printing Laser ablation	Young’s modulus, *E* ~ 3.1 GPa Low susceptibility to moisture Optically transparent	Micro-channel [189] Acceleration sensor [190] Nano-structure arrays [191]

Various polymers were demonstrated as alternative materials to solid-state semiconductors. However, SU-8 is found to be particularly suitable for MEMS applications. Variants of SU-8 polymers are classified primarily based on their viscosity and processing time, which include commercially available SU-8 2, SU-8 5, SU-8 10, SU-8 25, SU-8 50, SU-8 100, etc. However, new generation of SU-8 2000 series polymers are now widely used due to their better adhesion properties, improved coating, and faster processing time, especially for realizing piezoresistive SU-8 cantilever sensors. SU-8 polymers have advantages such as ability to form high-aspect-ratio and mechanically stable structures, inertness to chemicals, and compliance to fabrication facilities and techniques used in conventional IC fabrication processes like photolithographic process, dry or wet etching. Photosensitivity of SU-8 polymer combined with the ability to produce sharp edges even for large thicknesses has enabled MEMS engineers to realize high-aspect-ratio structures (> 20) by using UV lithography [192]. Furthermore, due to advantages like higher refractive index, biocompatibility, and controllability over its electric, magnetic, optical, and mechanical properties, SU-8 has become the preferred polymeric material for realizing miniaturized devices. In SU-8 polymeric piezoresistive sensors, the piezoresistive element is either gold, doped polysilicon, or doped SU-8. To understand the importance of material selection in determining the *G*/*E* ratio, we have detailed the typical values (*G* and *E*) of materials which are used to realize piezoresistive cantilever sensors as summarized in Table 6. It is observed that all the solid-state semiconductor-based designs have *G*/*E* ratio less than the SU-8 polymer-based designs (except in the case of Au piezoresistor and SU-8 structural layer). The higher *G*/*E* ratio of polymer-based design (with doped polysilicon and doped SU-8) is attributed to the lower Young’s modulus of SU-8 structural layer. Although doped polysilicon and CB SU-8 piezoresistor-based SU-8 polymeric cantilevers depict high electrical sensitivity, SU-8 polymeric cantilevers with graphene nano-platelet piezoresistors have been reported to have sensitivity in parts per billion (ppb) concentrations in ambient conditions for detecting explosive 2, 4, 6-trinitrotoluene (TNT). Compared to other combinations of piezoresistor material and SU-8 structural layer, this ultra-sensitivity of graphene-based SU-8 polymeric cantilever is primarily due to the high gauge factor of the graphene nano-platelet piezoresistor (*G* = 144) [195].

**Table 6:** Various combinations of materials for piezoresistor and structural layer with their respective G/E ratios [90, 192–195]

Structural layer	Young’s modulus (*E*) (GPa)	Piezoresistor	Gauge factor (*G*)	*G*/*E*
Si	169	Si	140	0.82
SiO_2_	70	Si	140	2.0
	70	p-poly-Si	20	0.28
Si_3_N_4_	250	Au	2	8 × 10^−3^
	250	p-poly-Si	20	8 × 10^−2^
SU-8	5	Au	2	0.40
	5	p-poly-Si	20	4.0
	5	CB SU-8	20	4.0
	5	Graphene nano-platelet–SU-8	144	28.8

In recent years, much focus of both industry and academia has been on developing state-of-the-art SU-8 polymeric piezoresistive cantilever sensors. Researchers have explored various possibilities at material level, device level, and fabrication process optimization for developing systems with high performance-to-cost index. Recently, Adams et al. [196] demonstrated SU-8-based polymeric cantilevers depict 19 times higher imaging in-air detection bandwidth than their conventional counterparts for similar size and mechanical characteristics. With focus on performance optimization, materials like SU-8/ZnO nano-composite nano-wires have been investigated for realizing devices [197]. Highly conductive CB-doped SU-8 nano-composite at low percolation threshold with good mechanical strength and photopattern ability has been reported for realizing cantilever sensors [198]. Process parameter optimization has been also reported in the literature. For instance, optimization of baking temperatures and release methods has been reported for maximizing the fabrication yield [199]. It has been found that baking temperature influences deformation of fabricated SU-8 device, especially SU-8 cantilevers due to the residual stress component generated within the structure. In addition, out of three releasing methods (dry method—fluorocarbon film, and wet method—Omnicoat sacrificial layer and polymethyl methacrylate sacrificial layer), wet release method using polymethyl methacrylate sacrificial layer was found to give the highest yield of 90%. Using the optimized recipe, SU-8 cantilever aptasensors were demonstrated for thrombin detection.

At the system level, SU-8 cantilevers vertically allocated in micro-fluidic channel have been demonstrated with enhanced performance [200]. Conductive SU-8 nano-composite comprising silver nanoparticles have been demonstrated to realize electronics components and interconnect on flexible substrate for sensing application [201]. The reported miniaturized electronic components and high-density interconnects were realized using low-cost micro-fabrication techniques. Realization of such high-density electronic components at reduced cost compared to their semiconductor counterparts paves a way to realize homogeneous SU-8 polymeric devices and signal processing circuitry. Experimental results have been reported for developing miniaturized devices with low-cost fabrication process using SU-8 as sacrificial layer [202]. Research on the methods of immobilization has been also reported to improve the biological sensitivity without affecting the SU-8 cantilever structure. Typically, harsh chemical treatment during immobilization of receptor has a detrimental impact of device structure. Recently, a vapor phase deposition of self-assembled monolayers with reduced impact on device structure has been reported [203].

The performance optimization of cantilever sensors using various innovative designs and process optimizations has been also reported. Sensor performance optimization has been carried out by careful structural optimization [204–207] and material selection [208–210]. The performance of SU-8 polymeric piezoresistive micro-cantilever sensors is determined not only by electrical sensitivity governed by material parameters of piezoresistor gauge factor and Young’s modulus of structural layer, but also by geometrical factors and noises (both intrinsic and extrinsic). Geometrical parameters include the cantilever platform and piezoresistor shape (including constituent layer dimensions) and are governed by desired electrical sensitivity and mechanical stability of the sensor from external vibrational noises. In addition, the sensor design is also governed by noise sources like thermal drift of sensor output, invalid detection due to temperature variation of cantilever surface induced by either external temperature change or joule heating of the dc-excited piezoresistor, variation in sensor output due to change in pH of operational medium, plastic deformation and electro-migration (in case of metal piezoresistors) due to joule heating, change in cantilever resonant frequency due to moisture absorption by SU-8 polymer, biological noise floor due to non-specific target–receptor interactions, to mention a few. Importantly, the aforementioned factors are limited by fabrication constraints.

Primary reliability issues are mainly vulnerability of SU-8 cantilevers to moisture, resist aging and temperature sensitivity. Moisture absorption not only impacts the mechanical sensitivity, but also has a detrimental impact on piezoresistor stability when polymeric cantilevers are used in organic solvents and buffer solution. Similarly, temperature susceptibility results in invalid detection, thermal drift-induced inaccuracy in sensor output characteristics, and plastic deformation. Various solutions have been devised to overcome the aforementioned limitations. In-depth investigation on understanding the atomistic physics of moisture absorption and its impact on system level of micro-/nano-epoxy-bonded systems depicts the hierarchical structure of the epoxy-bonded system is crucial for the interfacial integrity [211]. Recently, a parylene-C-coated SU-8 cantilever has been reported with reduced moisture vulnerability and better stability [212]. Seldom researchers have addressed the self-heating effect on SU-8 polymeric piezoresistive micro-cantilever sensors. However, for solid-state semiconductor piezoresistive cantilever sensors, the literature encompasses a few examples which detail the impact of self-heating and provide design solutions to overcome the inaccuracy induced due to thermal drift [213, 214]. Such solutions can be also extended to SU-8 polymeric piezoresistive cantilever sensors. More specifics on the aforementioned parameters which determine sensor performance and their dependence on fabrication techniques adopted to realize the sensor are detailed in the subsequent sections.

## SU-8 Polymer-Based Piezoresistive Cantilever Sensors

**Fig. 14:**
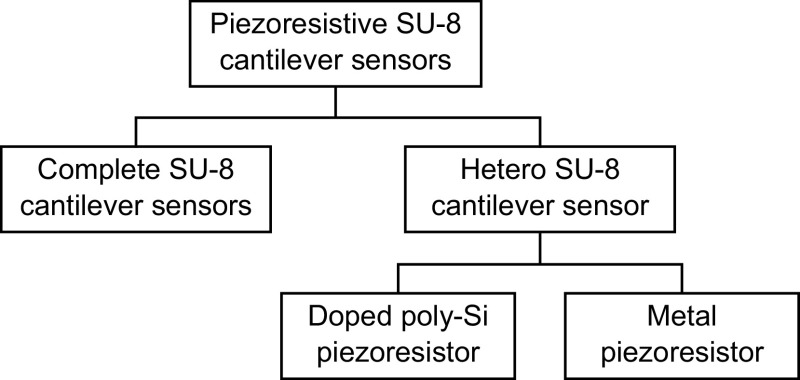
Classification of SU-8 piezoresistive cantilever sensors based on the piezoresistor material

SU-8 is an epoxy-acrylate-based negative-tone chemically amplified photoresist (PR) polymer which constitutes SU-8 monomers with 8 epoxy groups each forming the polymer matrix and comprising resin (SU-8 monomers), organic solvent, and photoacid generator. On exposure to light [215] or high-energy proton beam [216], SU-8 polymers undergo chemical amplifications that result in polymerization (cross-linking of monomers) due to photoacid generation. However, the complete cross-linking of monomers takes place only at elevated temperatures, which becomes essential to obtain mechanical stability. For more details on the variants of SU-8 polymers, their classification, and thermo-electromechanical characteristics readers may refer the literature [217, 218]. SU-8 polymers have been found versatile applications like optical waveguide [132], neural probe [172], micro-pump [219], micro-needle [173], micro-gripper [220], micro-gear [221], micro-mold [222], micro-lens array [223], micro-channel [224], AFM cantilever [175], micro-pillar [225], and micro-resonator [175]. In recent times, SU-8 polymers have also been extensively explored for realizing piezoresistive cantilevers for chemical and biological sensing. SU-8 piezoresistive cantilever sensors can be classified based on either geometrical features or constituent materials of the composite cantilever structure. Here, we have classified such sensors based on the piezoresistor material utilized to carry out the electromechanical transduction, as depicted in Fig. 14.

Based on the piezoresistor material, SU-8 polymeric cantilever sensors are classified into two types: hetero- and complete SU-8 polymeric cantilever sensors. While hetero-SU-8 cantilevers are designed with a piezoresistor material different from the SU-8 structural layer, the piezoresistors in complete SU-8 cantilevers are realized by electrically conductive SU-8 polymer itself. Further, the hetero-SU-8 cantilever sensors are categorized as metal and doped polysilicon piezoresistor-based sensors. It may be noted that the piezoresistive material not only determines the geometrical design aspects of SU-8 cantilever sensors, but also affects the fabrication process flow to realize such sensors.

Specifics of a few reported metal, doped polysilicon, and doped SU-8 piezoresistor-based SU-8 cantilever sensors detailing their type, cantilever stack configuration, cantilever shape, piezoresistor type, electrical and mechanical parameters are summarized in Table 7. The sensors reported vary in terms of the cantilever geometry, constituent layers, and piezoresistor material. Various cantilever shapes like rectangle (slender and wide), square, V shape with slit, and U shape have been used to realize piezoresistive SU-8 cantilever sensors. Irrespective of the piezoresistive element in SU-8 sensors, the structural layer and isolation layer are realized with SU-8 polymer. The sensors are realized with either three or four constituent layers. It may be noted that in three-layered structures, there is no separate immobilization layer and the SU-8 isolation layer itself is immobilized with receptors. However, to improve the specificity of detection, a separate Au immobilization layer is also incorporated by various researchers.

**Table 7:** Material, design, and performance details of piezoresistive SU-8 polymer cantilever sensors

Type	References	Cantilever stack		Shape	Dimensions	Electrical parameters	Mechanical parameters
		Piezoresistor	Other layers				
Hetero-polymeric cantilever	[87] (2002)	Au	Immobilization and isolation layers: SU-8, structural layer: SU-8	Rectangle	Cantilever: rectangle *L*_C_ = 200 µm, *W*_C_ = 100 µm, *t*_C_ = 7.6 µm, *t*_1_ = 1.8 µm, *t*_3_ = 5.8 µm Piezoresistor: meander-shaped *t*_2_ = 40 nm	Nominal resistance, *R* = 1.5 kΩ Δ*R*/*R* (nm)^−1^ = 0.3 × 10^−6^ Δ*R*/*R* (nm)^−1^ = 0.3 × 10^−4^ Bias voltage = 4.5 V	Spring constant = 7 N m^−1^ Resonant frequency = 49 kHz Deflection range = 0-60 µm Min. detectable *Z* = 4 Å Min. detectable *σ*_s_ = 1.4 × 10^−4^
Hetero-polymeric cantilever	[66] (2005)	Au	Immobilization: Au, isolation layer: SU-8, structural layer: SU-8 (2002)	Wide rectangle	Cantilever: wide rectangle *L*_C_ = 215 µm, *W*_C_ = 280 µm, *t*_4_ = 1.5 µm Piezoresistor: meander *t*_3_ = 60 nm, *W*_P_ = 4–6 µm	Nominal resistance, *R* = 500 Ω *V*_o_ = 80 µV at *σ*_s_ = 2 N m^−1^ and *Z* = 2 µm	*Spring constant = 0.31 N m^−1^ *Resonant frequency = 17.56 kHz *Δ*Z* (Nm)^−1^ = 9.61 nm
Hetero-polymeric cantilever	[226] (2006)	Au	Immobilization: Au, isolation layer: SU-8, structural layer: SU-8 (2002)	Wide rectangle	Cantilever: wide rectangle *L*_C_ = 215 µm, *W*_C_ = 280 µm, *t*_1_ = 20 nm and *t*_total_ ~ 3.5 µm Piezoresistor: meander *t*_3_ = 60 nm	Nominal resistance, *R* ~ 500 Ω *V*_o_ = 1 µV at *σ*_s_ = 20 × 10^−3^ N m^−1^ at *V*_b_ = 0.5 V	*Spring constant = 4.02 N m^−1^ *Resonant frequency = 40.99 kHz *Δ*Z* (Nm)^−1^ = 1.76 nm
Complete polymeric cantilever	[227] (2006)	CB SU-8	Immobilization and isolation layers: SU-8, structural layer: SU-8 (2002)	V-shaped with V-shaped slit	Cantilever: V-shaped *L*_C_ = 250 µm, *W*_C_ = 100 µm, *t*_3_ = 1.5 µm, and *t*_total_ ~ 3.5 µm Piezoresistor: V-shaped	Nominal resistance, *R* = 540 kΩ Δ*R*/*R* (nm)^−1^ = 1.12 × 10^−6^ Δ*R*/*R* (nm)^−1^ = 7.6 × 10^−3^ *V*_b_ = 0.5 V	*Spring constant = 0.91 N m^−1^ *Resonant frequency = 30.32 kHz *ΔZ (Nm)^−1^ = 2.38 nm
Complete polymeric cantilever	[228] (2006)	CB SU-8	Immobilization and isolation layers: SU-8, structural layer: SU-8 (2002)	Square	Cantilever: square *L*_C_ = 200 µm, *W*_C_ = 200 µm, *t*_1_ = 0.8 µm, *t*_3_ = 4 µm, and *t*_total_ ~ 7 µm Piezoresistor: *t*_2_ = 1.4 µm	Nominal resistance, *R* = 185 kΩ Δ*R*/*R* (nm)^−1^ ~ 3.2 × 10^−6^	*Spring constant = 28.58 N m^−1^ *Resonant frequency = 94.75 kHz *Δ*Z* (Nm)^−1^ = 0.38 nm
Hetero-polymeric cantilever	[229] (2006)	Au	Immobilization layer: Au, isolation layer: SU-8, structural layer: SU-8	Wide rectangle	Cantilever: wide rectangular *L*_C_ = 215 µm, *W*_C_ = 280 µm, *t*_1_ = 20 nm, *t*_2_ = 1 µm, *t*_4_ = 2.5 µm and *t*_total_ ~ 3.5 µm Piezoresistor: meander *L*_P_ = 100 µm, *t*_3_ = 60 nm	Nominal resistance, *R* = 600 Ω	*Spring constant = 4.02 N m^−1^ *Resonant frequency = 40.99 kHz *Δ*Z* (Nm)^−1^ = 1.76 nm
Hetero-polymeric cantilever	[230] (2009)	Doped poly-Si	Immobilization and isolation layers: SU-8 (2002), structural layer: SU-8 (2001)	U-shaped rectangle	Cantilever: U-shaped rectangle *L*_C_ = 200 µm, *W*_P_ = 40 µm, *t*_1_ = 0.9 µm, *t*_3_ = 1.8 µm Piezoresistor: U-shaped rectangle *L*_P_ = 200 µm, *W*_P_ = 40 µm, *t*_2_ = 120 nm	Nominal resistance, *R* = 110 kΩ Δ*R*/*R* (nm)^−1^ = 4.4 × 10^−6^	Spring constant = 0.31 N m^−1^ Resonant frequency = 39 kHz
Complete polymeric cantilever	[67] (2011)	CB SU-8	Immobilization layer: Au, isolation layer: SU-8, structural layer: SU-8	U-shaped	Cantilever: U-shaped *L*_C_ = 250 µm, *W*_P_ = 50 µm, *t*_1_ = 30 nm, *t*_2_ = 0.5 µm, *t*_4_ = 1.6 µm Piezoresistor: U-shaped *t*_3_ = 0.9 µm	Δ*R*/*R* (nm)^−1^ = 1.12 × 10^−9^ Min. detectable *σ*_s_ = 39 × 10^−3^ N m^−1^	Spring constant = 0.44 N m^−1^ Resonant frequency = 22.6 kHz
Complete polymeric cantilever	[192] (2011)	CB SU-8	Immobilization and isolation layers: SU-8 (2001), structural layer: SU-8 (2002)	Rectangle	Cantilever: rectangle *L*_C_ = 250 µm, *W*_C_ = 120 µm, *t*_1_ = 0.4 µm, *t*_3_ = 1.8 µm Piezoresistor: rectangle *L*_P_ = 80 µm, *t*_2_ = 0.8–1.2 µm	Resistivity, *ρ* = 2.7 Ω cm Δ*R*/*R* (nm)^−1^ = 0.55 × 10^−6^ Δ*R*/*R* (nm)^−1^ = 4.1 × 10^−3^	*Spring constant = 1.00 N m^−1^ *Resonant frequency = 29.45 kHz *Δ*Z* (Nm)^−1^ = 2.53 nm
Hetero-polymeric cantilever	[231] (2012)	Ti	Isolation layer: SU-8, structural layer: SU-8 (2000.5)	Rectangle	Cantilever: rectangle *L*_C_ = 500 µm, *W*_C_ = 200 µm, *t*_1_ = 0.5 µm, *t*_3_ = 11.4 µm Piezoresistor: U-shaped rectangle *L*_P_ = 400 µm, *W*_P_ = 40 µm, *t*_2_ = 0.1 µm	Resistivity, *ρ* = 1320 Ω nm (Δ*R*/*R*)/*ε* = 3.78 Δ*R*/*R* (nm)^−1^ = 0.18 × 10^−6^	*Spring constant = 8.98 N m^−1^ *Resonant frequency = 25.77 kHz *Δ*Z* (Nm)^−1^ = 0.82 nm
Complete polymeric cantilever	[232] (2012)	CB SU-8	Immobilization and isolation layers: SU-8 (2002), structural layer: SU-8 (2000.5)	V-shaped with V-shaped slit	Cantilever: V-shaped *L*_C_ = 200 µm, *W*_C_ = 100 µm, *t*_1_ = 1.6 µm, *t*_total_ ~ 3.5 µm Piezoresistor: V-shaped	Nominal resistance, *R* = 550 kΩ Δ*R*/*R* (nm)^−1^ = 1.12 × 10^−6^ Bias voltage = 0.5 V	*Spring constant = 1.78 N m^−1^ *Resonant frequency = 47.37 kHz *Δ*Z* (Nm)^−1^ = 1.52 nm
Complete polymeric cantilever	[233] (2014)	CB SU-8	Immobilization layer: Au, isolation layer: SU-8 (2002), structural layer: SU-8 (2000.5)	V-shaped cantilever with V-shaped slit	Cantilever: V-shaped *L*_C_ = 250 µm, *W*_C_ = 100 µm, *t*_1_ = 30 nm, *t*_2_ = 0.1 µm, *t*_total_ ~ 3.5 µm Piezoresistor: V-shaped	Nominal resistance, *R* = 520 kΩ Δ*R*/*R* (nm)^−1^ = 1.12 × 10^−6^ Δ*R*/*R* (nm)^−1^ = 7.6 × 10^−3^ Bias voltage = 0.5 V	*Spring constant = 0.91 N m^−1^ *Resonant frequency = 30.32 kHz *Δ*Z* (Nm)^−1^ = 2.38 nm
Complete polymeric cantilever	[234] (2015)	CB SU-8/CB SU-8 glycidol	Immobilization and isolation layers: SU-8 (2002), structural layer: SU-8 (2002)	U-shaped	Cantilever: U-shaped *L*_C_ = 250 µm, *W*_P_ = 50 µm, *t*_total_ ~ 3.5 µm Piezoresistor: U-shaped	Δ*R*/*R* (nm)^−1^ = 7.5 × 10^−7^	Spring constant = 0.37 N m^−1^ Resonant frequency = 16.09 kHz

*Computed values

Lateral dimensions of the cantilever platform and thicknesses of the constituent layers are designed to meet specifications of mechanical stability, i.e., spring constant, resonant frequency, and electrical sensitivity. To ensure stability and compliance of the cantilever, the spring constant is typically chosen in the range from 0.1 × 10^−3^ to 10 N m^−1^, whereas to reduce the vulnerability of the sensor from external vibrational noise, the sensor is designed with resonant frequency more than 5 kHz. The lateral dimensions of the cantilever are also governed by factors like piezoresistor coverage area, sensor die size, etc. Compared to the typical thickness of solid-state semiconductor cantilevers (less than 1 µm), the thickness of SU-8 cantilevers is kept more than 1 µm mainly to ensure mechanical stability of the cantilever platform. It may be noted that in all the devices mentioned, careful design is performed such that the distance between the mid-plane of piezoresistor and the neutral axis of the cantilever stack is maximum, thereby maximizing the electrical sensitivity. For instance, in most cases, the thickness of the structural layer is kept more than three times the isolation layer thickness. Even though a separate Au immobilization layer is used, its thickness is kept minimal (nm) to maximize electrical sensitivity.

Piezoresistor geometry is chosen based on the desired nominal resistance value and signal-to-noise ratio (SNR). Lower limit of nominal resistance is determined by joule heating-induced self-heating effects and electro-migration, whereas its upper limit is determined by specifications of driving current and interface circuitry. Thus, typically the value of nominal resistance of piezoresistors is kept in kΩ range. In general, the piezoresistors are designed to be U-shaped to improve reliability by avoiding interconnects on the cantilever platform. Since the electrical resistivity of metals is low (in the range of µ Ω-cm), when metal piezoresistors are designed in U shape, their nominal resistance is only a few ohms, which results in large joule heating. Thus, strategically the metal piezoresistors are designed lengthier to obtain large nominal resistance. Therefore, in few cases, rather than the conventional U-shaped design, the piezoresistors are meander-shaped. However, the coverage of the piezoresistor is limited by the cantilever lateral dimensions, since lengthier cantilever platforms result in reduced mechanical stability and electrical sensitivity. On the other hand, by tailoring the electrical resistivity of the piezoresistor material and careful design, it is possible to realize U-shaped metal piezoresistors. Similarly, the width and thickness of the piezoresistor are designed to obtain a desirable nominal resistance. Thickness of metal piezoresistors has an additional constraint on noise figure, since thinner metal piezoresistors show higher electrical noise level. The need for high nominal resistance value to reduce the current density and therefore the joule heating effects is achieved by using lower supply voltages. In the case of doped polysilicon or doped SU-8 piezoresistors, the electrical resistivity is controlled by varying the dopant concentration. Thus, by optimizing the dopant concentration and geometry of the piezoresistor, desired nominal resistance is achieved. Typical magnitude of surface stress sensitivity and deflection sensitivity of SU-8 piezoresistive cantilever sensors are in the range of a few × 10^−3^ (N/m)^−1^ and a few × 10^−6^ to × 10^−9^ (nm)^−1^ which can detect miniscule forces of pN magnitude and lower.

Comprehensive specifics of the metal, doped polysilicon, and doped polymer-based SU-8 cantilever sensors are detailed in the subsequent sections.

### Hetero-SU-8 Polymeric Cantilevers

In SU-8-based hetero-polymeric cantilever sensors, the structural and isolation layers are realized with SU-8 polymer, whereas the piezoresistor is either a metal or a semiconductor element.

#### Cantilevers with Metal Piezoresistors

The combination of metal piezoresistor (Au) with SU-8 polymer structural layer was first demonstrated by Thaysen et al. in 2002 [87]. Under stress, unlike semiconductor piezoresistors in which deformation of energy bands results in change in electrical resistivity, in metal piezoresistors geometrical variations (strain) cause the resistance to change. Over the years, various metals and their alloys have been investigated for application in strain sensing which includes titanium (Ti) [231], gold (Au) [235], copper (Cu) [236], bismuth–antimony (Bi–Sb) [237], copper–nickel (Cu–Ni) constantan alloy [238], nickel–chromium (Ni–Cr) [239], palladium–chromium (Pd–Cr) [240], platinum (Pt) [241], manganese (Mn) [242], and nickel–silver (Ni–Ag) [243].

**Fig. 15:**
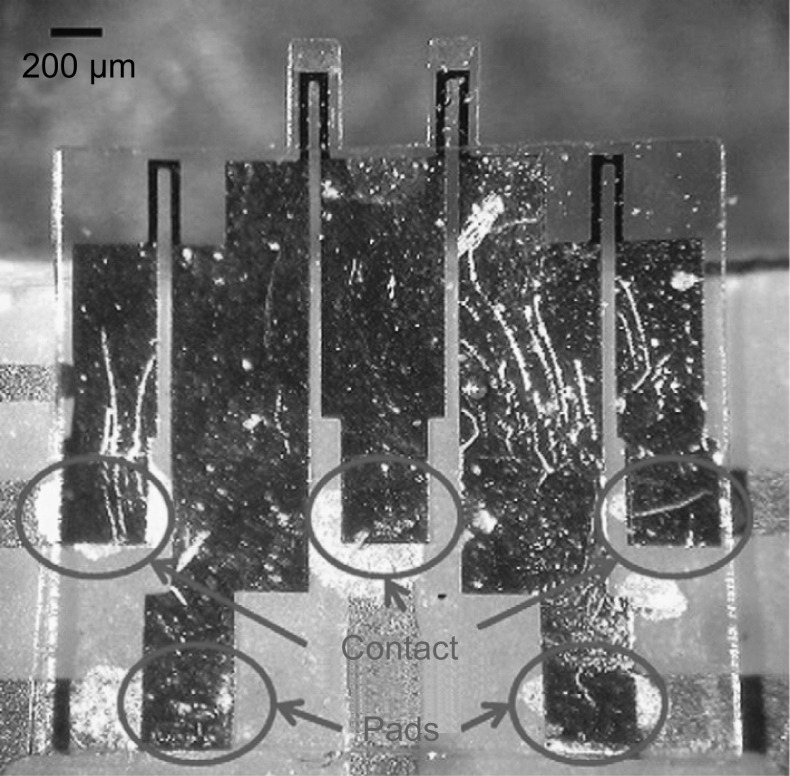
Optical image of U-shaped titanium piezoresistor-based SU-8 cantilever sensors operated in differential mode with passive resistors embedded in WSB configuration. Adopted from Ref. [231]. Copyright (2012) Elsevier B.V.

In metal piezoresistor-based cantilever sensors, a metal resistive layer is deposited atop the SU-8 structural layer. In order to prevent direct contact between the external environment and the electrically active metal piezoresistor, a thin layer of SU-8 is coated over the metal resistive element. Metal piezoresistors have the inherent advantage of low Johnson and 1/*f* noise [244]. Moreover, the sensitivity factor (G/E—ratio of gauge factor of the piezoresistor to the Young’s modulus of the structural layer) of metal, especially Au- and Ti-based SU-8 cantilever sensors, is better than the combination of metal or doped polysilicon piezoresistor-based cantilevers with structural layer realized with other materials. An optical image of U-shaped metal (Ti) piezoresistor-based SU-8 cantilever sensor is shown in Fig. 15. The image shows two micro-cantilever sensors with U-shaped integrated piezoresistors connected in a Wheatstone bridge (WSB) configuration with two on-chip resistors. The differential measurement results not only in the reduction in external noises from the environment like mechanical vibrations, but also in cancellation of internal noise factors like resistor mismatches and thermal drift [245].

Even though metal piezoresistor-based SU-8 cantilevers are used as an alternative for solid-state semiconductor-based design, they suffer from a few limitations. It is preferred to design cantilevers with high value of nominal resistance of the piezoresistor primarily to reduce the magnitude of current and therefore the resulting Joule heating. However, it may be noted that due to very low resistivity of metals, the maximum value of nominal resistance of metal piezoresistors cannot be increased beyond a certain limit. Thus, when biased with a dc source compared to other piezoresistive materials, metal piezoresistors carry large volumetric current that results in significant Joule heating. The Joule heating of the metal piezoresistor combined with lower thermal conductivity of SU-8 polymer matrix results not only in the difference in TCE-induced cantilever deflection, but also in an increase in Johnson noise floor and plastic deformation of the cantilever. Increasing the piezoresistor length is an option to increase the nominal resistance value. However, this adversely affects the electrical sensitivity of the device as a major portion of the resistor may be placed on the unstressed region of the cantilever. Other option is to design serpentine-shaped metal piezoresistors and biasing it with low dc voltage. Apart from the aforementioned factors, electro-migration is also closely related to the high magnitude of current density and, more importantly, the miniaturized size of the piezoresistor leading to instability in its resistance value [87]. Adhesion issue of metal with polymer is another issue that can lead to device failure. Designs such as the sensor reported in Ref. [231], where researchers have implemented titanium metal piezoresistor that does not require any adhesive layer.

In summary, although metal piezoresistors depict appreciable value of G/E ratio, they suffer from limitations such as Joule heating-induced inaccuracies, instability of resistance value due to electro-migration, and adhesion issues with SU-8 polymer.

#### Cantilevers with Polysilicon Piezoresistors

An alternative piezoresistive material to metal piezoresistor is doped polysilicon. In polysilicon piezoresistor-based SU-8 cantilever sensors, a thin film of polysilicon is used as the piezoresistor material. Even though doped polysilicon piezoresistors along with SU-8 polymeric structural platforms have been demonstrated to have better performance (G/E) than metal-based piezoresistors, realization of such sensors is limited mainly due to the following reasons: (1) vulnerability of SU-8 polymers to high-temperature deposition processes such as low-pressure and plasma-enhanced chemical vapor deposition (CVD) to realize polysilicon piezoresistors, and (2) reduction in electrical sensitivity due to higher stiffness of sensor structure with an integrated polysilicon piezoresistor. Limitations due to cantilever stiffness can be overcome by careful design and dimensional optimization of sensors [246].

**Fig. 16:**
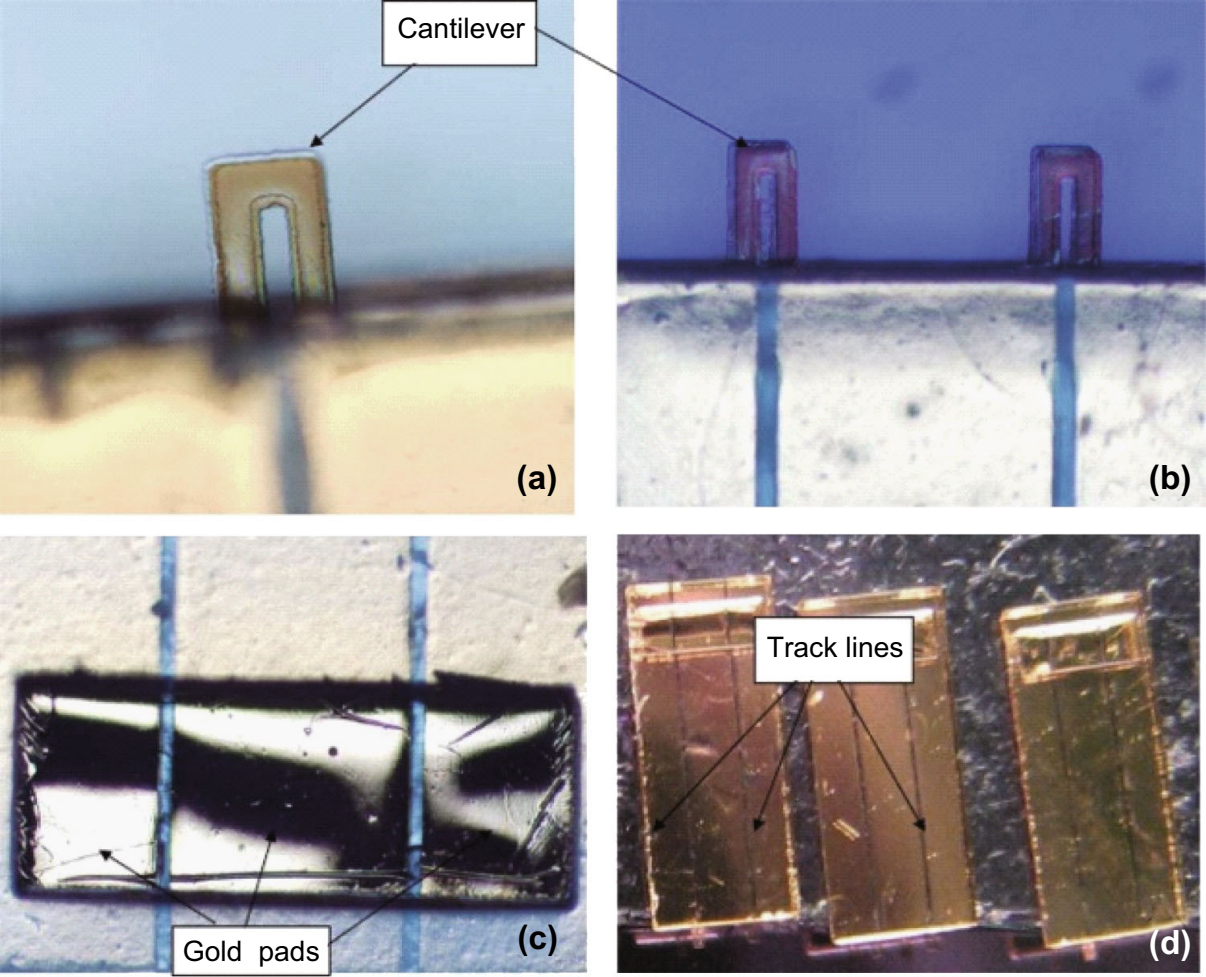
Optical images of **a** a U-shaped polysilicon piezoresistor-based SU-8 cantilever sensor, **b** two U-shaped cantilever sensors, **c** gold pads and track lines, and **d** complete die. Adopted from Ref. [230]. Copyright (2009) IEEE

Electrical properties of polysilicon are a strong function of its grain size, characteristics of grain boundaries, crystal orientation, doping type, and concentration [247]. More importantly, variation in the impurity concentration and process parameters can be used to tailor the electrical properties of polysilicon, especially the magnitude of the piezoresistive coefficients and the temperature coefficient of resistance (TCR) [248, 249]. Even though polysilicon has lower gauge factor compared to monocrystalline silicon-based piezoresistor, controllability over its TCR, lower transverse piezoresistive coefficients which become critical to realize surface stress-based sensors, and piezoresistive coefficients prove critical to overcome thermal drift in cantilever sensors. Polysilicon piezoresistors are realized with a low-temperature deposition technique known as hotwire chemical vapor deposition (HWCVD) [230]. Polysilicon piezoresistor is deposited atop the SU-8 structural layer and encapsulated by a thin coating of SU-8 isolation layer. The thin SU-8 layer atop the piezoresistor element acts as both the isolation and immobilization layers for target–receptor interactions. Optical images of the polysilicon piezoresistor-based U-shaped SU-8 cantilevers are shown in Fig. 16.

Despite various advantages, the performance of doped polysilicon-based SU-8 cantilevers is curtailed by the increased stiffness of composite cantilever structure when polysilicon piezoresistor is incorporated within. This is mainly due to the relatively higher Young’s modulus of polysilicon piezoresistor compared to SU-8 polymer [246]. A potential solution is to reduce the thickness of the piezoresistor, however at the cost of increased electrical noise.

### Complete SU-8 Polymeric Cantilevers

**Fig. 17:**
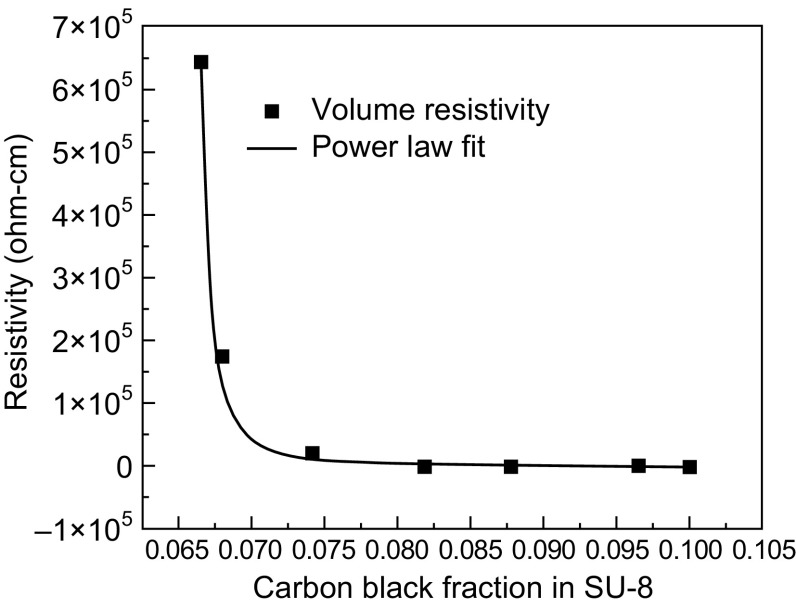
Variation in electrical resistivity of CB SU-8 composite as a function of CB doping. Adopted from Ref. [192]. Copyright (2009) Elsevier Masson SAS

In complete SU-8 polymeric cantilevers, all the constituent layers of the device are realized using SU-8 polymers. Here, rather than metal or doped polysilicon, conductive polymers are used as the piezoresistive material. In its native state, polymer matrix behaves as an electrical insulator. However, polymers are made conductive by a controlled addition of conducting nanoparticles known as conductive fillers. Treatise encompasses various examples of conductive fillers, which are physically dispersed in the polymer matrix to realize conductive polymers like carbon black (CB) [250], silver nanoparticles [251], copper [252], multi-walled carbon nanotubes (MWCNTs) [253], diamondoids [254], single-walled carbon nanotubes (SWCNTs) [254], and gold nanoparticles [254]. The degree of electrical conductivity of polymers added with conductive fillers is determined by the density of conductive nanoparticles and their distance in the polymer matrix. The dominant mechanism of electrical conduction is electron tunneling between the nanoparticles through the polymer film boundary [255]. In-depth specifics of the electric conduction mechanism and the factors that govern the electrical conductivity in conductive polymers are detailed in [256–259]. A typical example of variation in electrical resistivity of CB SU-8 composite as a function of CB doping in SU-8 matrix is shown in Fig. 17.

**Fig. 18:**
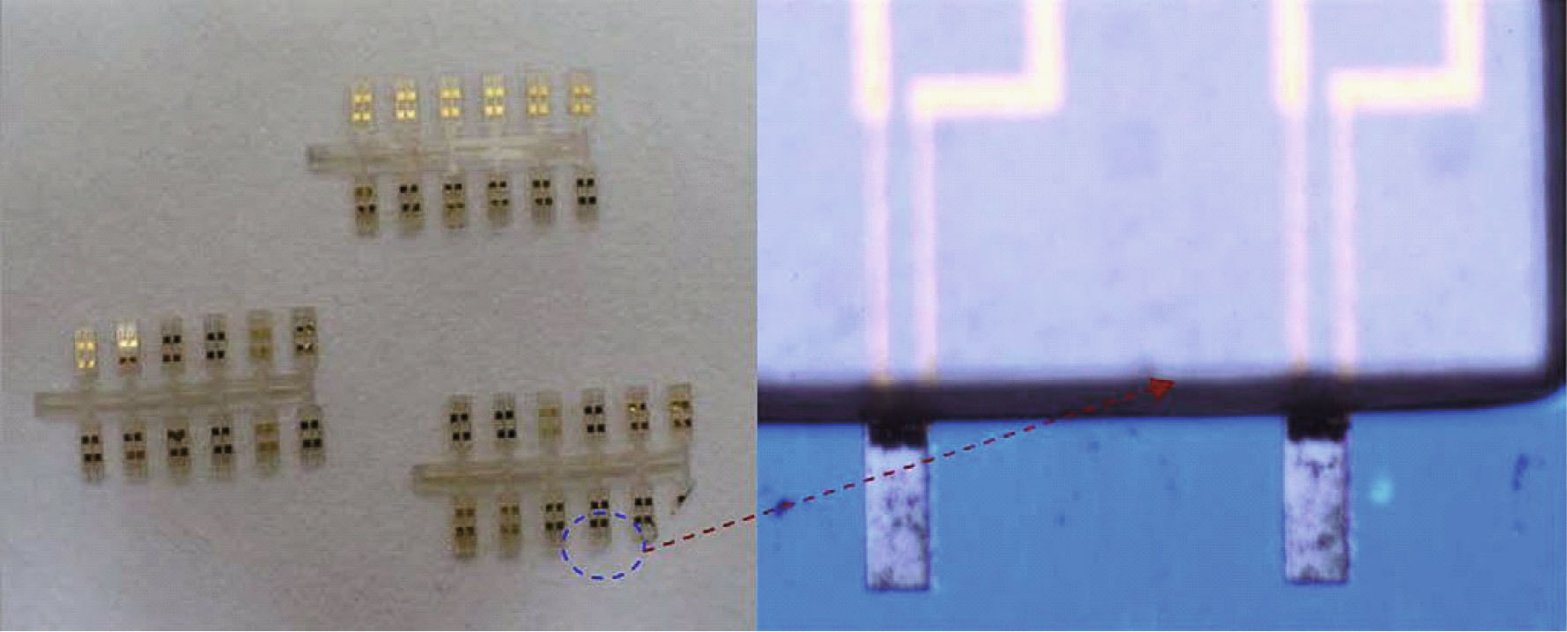
Optical image of CB-doped SU-8 piezoresistor-based SU-8 cantilever sensor arrays with a zoom-in image of a pair of cantilevers. Adopted from Ref. [192]. Copyright (2009) Elsevier Masson SAS

Complete SU-8 polymeric cantilevers overcome the limitations of increased stiffness of hetero-SU-8 polymeric cantilevers due to the integration of metal/polysilicon piezoresistors with higher Young’s modulus in the SU-8 cantilever stack. The conductive SU-8 polymer layer is spin-coated on the cantilever structural layer and then encapsulated with an isolation layer. The conductivity of doped composite SU-8 polymer depends mainly on the loading of conductive nanoparticles, dispersion and alignment of nanoparticles in the SU-8 polymer matrix, and the percolation limits. When cantilever undergoes deflection, the conductivity of CB-doped SU-8 piezoresistor changes due to distortions in the conductive network of dispersed nanoparticles in the polymer matrix. The optical image of a CB-doped SU-8-based piezoresistive cantilever sensor array is shown in Fig. 18. The higher gauge factor of CB-doped SU-8 and lower Young’s modulus of SU-8 structural layer result in G/E close to 4. In recent times, CB-doped SU-8 polymeric cantilever has been demonstrated to have better electrical sensitivity than optics-based designs [233].

## Fabrication Details of Piezoresistive SU-8 Polymeric Cantilevers

In this section, we elucidate the fabrication details of SU-8 piezoresistive cantilever sensors with metal, doped polysilicon, and CB-doped SU-8 polymer as the piezoresistor element. Unlike solid-state semiconductor piezoresistive sensors, which are predominantly realized by bulk micro-machining and require costly equipment like ion implantation machine and stringent clean room facilities, fabrication of SU-8 cantilevers includes steps like spin coating, deposition, and etching, which are relatively cost-effective in terms of both material and amenities required. In the subsequent sections, we detail the fabrication steps required to realize both hetero- and complete piezoresistive SU-8 polymeric cantilever sensors.

**Fig. 19:**
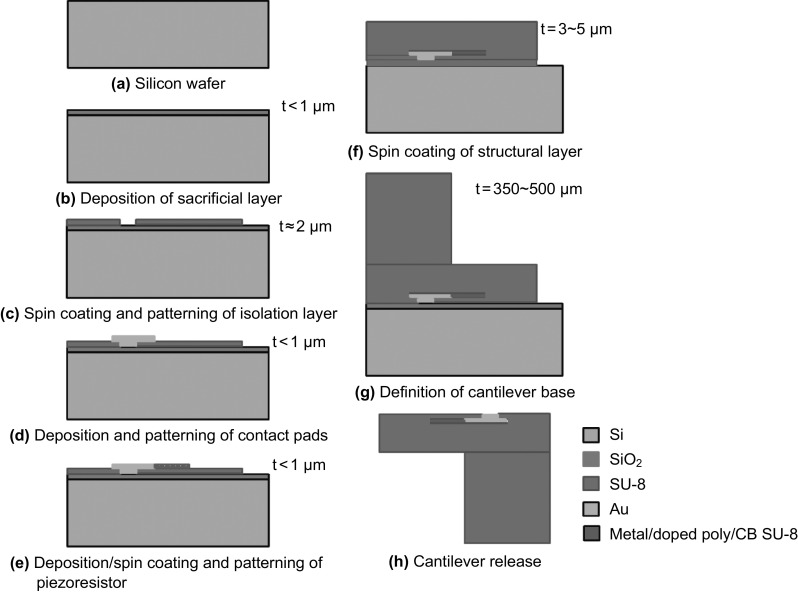
Generic fabrication process steps to realize metal/doped polysilicon/CB SU-8 piezoresistor-based SU-8 polymeric micro-cantilever sensor

A generic fabrication process flow used to realize metal/doped polysilicon/CB-doped SU-8 piezoresistor-based SU-8 polymeric cantilever sensors is shown in Fig. 19. The substrate wafer used as a base to realize the cantilevers is either an *n*- or *p*-type SCS wafer as shown in Fig. 19a. Post-realization of the SU-8 cantilevers, the base SCS wafer is separated from the cantilever structures and reused again. A thin sacrificial layer of Cr/Au/Cr in the case of metal piezoresistors, and SiO_2_ in the case of doped polysilicon and CB SU-8 piezoresistors, is deposited atop the SCS wafer as shown in Fig. 19b. Subsequently, in the next step, a relatively thin layer of SU-8 (thickness ≈ 2 µm) is spin-coated and patterned as depicted in Fig. 19c. This thin SU-8 layer serves as the isolation layer and protects the piezoresistor. Thickness of the spin-coated isolation layer plays a critical role in tailoring the distance between the mid-plane of the piezoresistor and the neutral axis of the cantilever stack, and therefore the electrical sensitivity. In the following step, a metal layer (with thickness < 1 µm) is deposited atop the thin SU-8 isolation layer and patterned with standard photolithographic process to define the contact pads as depicted in Fig. 19d. Then, a thin layer of piezoresistor (metal by sputtering/doped polysilicon by HWCVD/CB SU-8 by spin coating) is deposited and photolithographically patterned to define the piezoresistor element (as shown in Fig. 19e). Subsequently, a thick layer of SU-8 layer (thickness = 3–5 µm) is spin-coated and patterned to form the structural layer of the cantilever sensor (as depicted in Fig. 19f). To maximize electrical sensitivity, spin-coated SU-8 structural layer thickness is chosen higher than that of the SU-8 isolation layer. In the following step, a SU-8 polymer variant with high viscosity is spin-coated. This thick film of SU-8 (thickness = 350–500 µm) is patterned by a photolithographic process to realize the cantilever base as shown in Fig. 19g. Finally, the cantilever structure is released by etching the sacrificial layer using wet etching. More specifics of fabrication sequences and process parameters for realizing metal piezoresistor SU-8 sensors are detailed in Ref. [87, 231], whereas doped polysilicon and CB SU-8-based cantilever sensors are reported in Ref. [230, 260] and [217, 218, 233], respectively. For comprehensive details regarding the soft bake, exposure time, post-exposure bake, and processing of SU-8 thin film, readers are encouraged to refer the literature [261–265]. A summary of the piezoresistor materials and their corresponding fabrication techniques along with their features is summarized in Table 8.

**Table 8:** Various piezoresistor materials and their respective features used to realize SU-8 piezoresistive cantilever sensors

Piezoresistor	*G*/*E* ratio	Nominal resistance	Fabrication steps to realize the piezoresistor	Issues/features	References
Metal	0.4	500 Ω–1.5 kΩ	Sputtering/thermal evaporation	Higher rate of joule heating Plastic deformation of the cantilever Reduced SNR Electro-migration effect in resistors Adhesion of metal with SU-8	[68, 87, 226, 229, 231]
Polysilicon	4.0	100–200 kΩ	HWCVD	High dependence of electrical properties of poly-Si on process parameters Higher stiffness compared to metal piezoresistor Adhesion of poly-Si with SU-8	[217, 230, 260]
CB SU-8	4.0	185–550 kΩ	Spin coating	Higher gauge factor, low-temperature process Lower residual stress Dependence of mechanical properties on CB loading Electrical resistivity variation due to CB dispersion issues	[67, 192, 233, 250]

Various issues related to the fabrication of metal, doped polysilicon, and CB SU-8-based SU-8 cantilever sensors.

Challenges to realize metal piezoresistor-based SU-8 cantilever sensors include the following: (1) Cantilever release using Cr/Au/Cr sacrificial layer with wet etching process results in complexities. In the case of Cr/Au/Cr sacrificial layer, the first layer of Cr acts as an adhesive layer for Au, whereas the subsequent Au–Cr combination forms a galvanic cell which promotes faster etching [266]. However, the wet release of cantilever suffers from limitation due to the stiction [260] and lower etch rates of wet etchants. A potential solution of this problem was reported by Haefliger et al. [267], who demonstrated an innovative dry release technique with thin fluorocarbon film (an anti-stiction and hydrophobic coating) as the sacrificial layer. (2) Adhesion-related issues of metal and SU-8 polymer structure. (3) Deposition of metal on SU-8 results in residual stress-induced cantilever bending that leads to device failure [268].

Challenges to realize doped polysilicon piezoresistor-based SU-8 cantilever sensors include adhesion of polysilicon to SU-8, increase in the stiffness of cantilever stack due to higher Young’s modulus of polysilicon piezoresistor, and difficulty in controlling the process parameters of deposited polysilicon.

Challenges to realize doped SU-8 piezoresistor-based SU-8 cantilever sensors are given below: (1) Poor dispersion of conductive nanoparticles in the SU-8 polymer matrix results in reduced conductivity and control over the electrical resistivity of conductive polymer. The dispersion of conductive nanoparticles is improved by using a nano-thinner as demonstrated by Seena et al. [192] who used a nano-thinner 2000 for improving the dispersion of CB in SU-8 2002. (2) The mechanical characteristics like Young’s modulus of polymer with low concentration of doping (*E*_CB-SU-8_ approximately equals to 5 GPa) remain mostly unaltered [250]. More recently, it was shown that the CB-filled SU-8 matrix had increased by 30–40% for CB doping of 8 vol% [67]. (3) Variation in photosensitivity of SU-8 due to physical dispersion of nanoparticles directly affects the curing times and hence the quality of micro-fabrication.

Compared to metal and doped polysilicon-based piezoresistors, the spin-coated CB-doped piezoresistor overcomes the limitations of higher-temperature deposition techniques. In addition, since (conductive) SU-8 itself is used as the piezoresistor, the limitation of residual stress generated due to two dissimilar materials, which are found between metal–SU-8 and polysilicon–SU-8 cantilever platforms, is overcome. However, tight control over the process parameters becomes essential, especially to realize SU-8 as the piezoresistor element. Furthermore, since the polymer matrix absorbs water from its surroundings SU-8 is susceptible to humidity [269–272]. The absorption of humidity results in variations in the resonant frequency of SU-8 polymeric cantilevers, which affects the mechanical stability of the sensor [273]. However, humidity-related drifts and resist aging-related issues can be reduced by process optimization [66, 274]. Despite the aforementioned issues, SU-8 polymer-based piezoresistive cantilever sensors have been used for various chemical and biological sensing applications through careful design and process optimization.

## Applications

Piezoresistive SU-8 cantilever platform sensing technology combined with the advances in recognition techniques has been used for various chemical and biological sensing applications, as summarized in Table 9.

**Table 9:** Typical chemical and biological sensing applications of piezoresistive SU-8 polymeric cantilever sensors

References	Authors	Application
[87]	Thaysen et al.	Biochemical sensing
[230]	Kale et al.	Antigen–antibody (myoglobin)
[192]	Seena et al.	Explosive detection
[233]	Patil et al.	Explosive detection (TNT, RDX, and PETN)
[232]	Reddy et al.	Carbon monoxide (CO)
[67]	Seena et al.	Explosive detection (TNT)
[229]	Johansson et al.	DNA hybridization
[227]	Patil et al.	Humidity and moisture detection
[68]	Johansson et al.	Mercaptohexanol
[226]	Johansson et al.	Mercaptohexanol

Typical biological sensing applications include usage as a sensing and investigation tool in the field of biomedical engineering, especially genomics and proteomics. Recently, detection of biochemical entities has been also reported using SU-8 piezoresistive cantilevers. SU-8-based piezoresistive cantilever sensors capable of sensing surface stress in the range of few mN m^−1^, which is typical in the case of interaction of biochemicals on a gold surface, have been demonstrated by Thaysen et al. [87]. A specific example of biochemical sensing application is the detection of antigens like myoglobin (a cardiac disease marker). Myoglobin is a potential indicator of heart attacks in human, and its detection in human blood above a certain threshold could result in the early detection of heart attack and its prevention. Kale et al. [230] reported doped polysilicon-based SU-8 cantilevers, which exhibited sensitivity in the range of mN m^−1^, are capable of detecting myoglobin and anti-myoglobin interactions. Apart from detection of biochemicals, detection and investigation of human DNA was also demonstrated with surface stress detection sensitivity of a few mN m^−1^ using Au piezoresistor SU-8 cantilever sensors [229].

Detection of explosives like 2, 4, 6-trinitrotoluene (TNT), pentaerythritol tetranitrate (PETN), and hexahydro-1,3,5-triazine (RDX) becomes critical for homeland security mainly tackling chemical warfare. Compared to conventional explosive detection schemes like mass spectroscopy and ion mobility spectrometry (IMS), piezoresistive SU-8 cantilever sensors have advantages of compactness, faster response, better sensitivity, low sample volume, especially for detecting explosive in vapor phase, apart from the added benefits of MEMS technology. Typically, detection of nitro aromatic explosives like TNT, RDX, PETN is performed by immobilizing 4-mercaptobenzoic acid (4-MBA) or 6-mercaptonicotonic acid (6-MNA) receptors on Au surface using thiol immobilization protocol. When exposed to TNT molecules, such receptor molecules form hydrogen bonds with TNT, resulting in the change in surface stress. Piezoresistive SU-8 cantilever sensors have been demonstrated to detect TNT down to parts-per-billion [67, 192] and parts-per-trillion [233] concentration in vapor phase with excellent selectivity. In addition, detection of chemicals like mercaptohexanol has been also reported in the literature [68, 226].

In addition, piezoresistive SU-8 cantilever sensors have been used as artificial nose for environment monitoring applications such as detection of gases like carbon monoxide (CO) and moisture. Detection of gases like CO becomes essential due to its toxic nature and possible health hazards. Similarly, detection of moisture becomes important in fields like food storage and processing to prevent food spoilage. Piezoresistive SU-8 cantilevers coated with Fe(III) porphyrin have been demonstrated to detect CO gas with high specificity and sensitivity down to ppm [232]. Similarly, SU-8 cantilevers coated with polyaniline (PANI) nano-fibers showed good sensitivity toward moisture [227].

## Challenges and Future Perspectives

In the last decade, piezoresistive SU-8 cantilevers have been extensively explored as sensing platforms for detecting chemical and biological analytes. However, the transition of piezoresistive SU-8 cantilever sensors from a proof of concept to a potential replacement of conventional diagnostics techniques is limited by various challenges.

When used as a sensing tool, a SU-8 cantilever is functionalized with receptors for detecting a specific entity or analyte in a test sample. However, the test sample is a mixture of different entities with varying concentration. Typically, other entities are present in much higher concentration than the target molecules. Thus, the probability of non-specific interactions on the sensor surface is higher, which results in a significant biological noise floor. Moreover, specificity of such sensors becomes critical in determining the desired signal baseline. Premise is primarily influenced by the immobilization protocol adopted that determines the immobilization surface and target–receptor conjugate. Other sources that contribute to biological noise floor include the presence of more than one conjugate pair for the receptor molecules, non-uniform immobilization of receptors, and negligence in immobilization surface preparation. Non-specific interactions can be reduced by considering an immobilization material that is chemically different from SU-8 and by tight control over immobilization technique.

Piezoresistive SU-8 cantilever sensors with Au immobilization layer depict better performance metrics (electrical sensitivity and specificity) compared to sensors with SU-8 as the immobilization surface. However, incorporating Au immobilization layer in the cantilever stack increases the net TCE difference in the multilayer structure, thereby enhancing TCE-induced thermal drift in the sensor terminal characteristics. TCE-induced cantilever deflection becomes a serious problem, especially when metal piezoresistors and bias voltage more than 1 V are used. The aforementioned factors combined with low thermal conductivity of SU-8 polymers result in sensor failure due to plastic deformation of cantilever structure. Possible solutions to overcome thermal drift include direct immobilization of SU-8 surface, lower bias voltage, and complete polymeric cantilevers. However, for maintaining high degree of specificity and sensitivity, thickness and coverage of Au immobilization layer can be carefully optimized to minimize TCE-induced deflection.

A possible solution to avoid failure due to joules heating-induced plastic deformation of the cantilever is to adopt complete SU-8 polymeric cantilevers with conductive SU-8 as the piezoresistive element. However, controlling the electrical resistivity of conductive nanoparticles-dispersed SU-8 polymer matrix still remains a challenging task. Better controllability over the dispersion of nanoparticles can be obtained by tight control over process parameters and developing better percolation models to understand the phenomenon of electric conductivity in nanoparticles-dispersed polymer matrix.

The performance of piezoresistive SU-8 cantilever sensors is also influenced by external factors like humidity and temperature. When operated in controlled environment along with careful design for temperature compensation, the impact of temperature on sensor performance can be nullified. However, susceptibility of SU-8 polymers to humidity is a major issue. This problem becomes critical in SU-8-based chemical and biological sensors, since the operational environment in such sensors is predominantly liquid or vapor. Absorption of water or moisture changes the mass of SU-8 matrix and therefore directly affects the mechanical stability of the sensor. Vulnerability of SU-8 sensors to moisture can be reduced by optimizing the baking time of SU-8 polymers. Apart from the influence of external factors, SU-8 material and processing-related challenges like aging of SU-8 resist over a period of time, cracks in SU-8 films during processing, non-uniform exposure-induced dimensional inaccuracy of high-aspect-ratio structures, and delamination of SU-8 film from the substrate due to difference in TCE fabrication also induce failure.

Aging of SU-8 polymeric cantilevers is a cumulative effect of environmental factors like temperature, humidity, light with time. Aging of SU-8 polymer has an adverse impact not only on sensor reliability in terms of measurement, but also on sensor durability or life time. Aging over a period of time to humidity and temperature becomes more critical in the case of chemical and biological sensor due to continuous/cyclic exposure to moisture and temperature variation. However, the literature encompasses examples where researchers have performed extensive research to reduce the vulnerability of SU-8 polymeric cantilever to aging by optimization of process parameter (hard bake temperature) [274], solvent content [261], and exposure time [261]. It has been reported that by optimizing process parameters, SU-8 polymeric cantilevers can be used for months (approximately 200 days) without large deviation in initial deflection (with an error within 1°) [274].

Despite the aforementioned issues, compared to conventional solid-state semiconductor piezoresistive sensors, SU-8 polymer-based sensors have advantages in terms of reduced material and fabrication cost. In addition to high performance-to-cost ratio, SU-8 polymers have the advantage of biocompatibility, thus making them a preferred choice in chemical and biological sensing applications. Solutions to existing problems and further advancements in piezoresistive SU-8 cantilever sensors into a universal sensing tool would require close collaboration of researchers from science, engineering, and medicine streams.

## Conclusions

Piezoresistive SU-8 polymeric cantilever sensors emerged as one of the potential replacements for solid-state semiconductor-based designs mainly due to their high performance-to-cost ratio. The research and development of SU-8 polymer-based sensors has been a vibrant field. Continuous endeavor of researchers in developing piezoresistive SU-8 polymeric cantilever sensors has made cantilever platform a universal sensing tool for chemical and biological sensing applications. In this article, we have summarized the developments in piezoresistive SU-8 cantilever sensors and critically analyzed their performance considering the material selection, design, and fabrication aspects, and their interdependence. A brief insight into various theories of surface stress generation is detailed. Composite piezoresistive SU-8 micro-cantilever sensors have been classified as hetero- and completely polymeric cantilever. Specifics related to material selection, design, and fabrication of the aforementioned types of SU-8 polymeric cantilevers along with their performances are elucidated in detail. In addition, a critical comparison of the performance of piezoresistive SU-8 cantilever variants with their solid-state semiconductor counterparts using analytical models is also made. Finally, we cited existing challenges and various applications of piezoresistive SU-8 cantilever sensors as a chemical and biological sensing platform.
